# Standardizing Flow Cytometry Immunophenotyping Analysis from the Human ImmunoPhenotyping Consortium

**DOI:** 10.1038/srep20686

**Published:** 2016-02-10

**Authors:** Greg Finak, Marc Langweiler, Maria Jaimes, Mehrnoush Malek, Jafar Taghiyar, Yael Korin, Khadir Raddassi, Lesley Devine, Gerlinde Obermoser, Marcin L. Pekalski, Nikolas Pontikos, Alain Diaz, Susanne Heck, Federica Villanova, Nadia Terrazzini, Florian Kern, Yu Qian, Rick Stanton, Kui Wang, Aaron Brandes, John Ramey, Nima Aghaeepour, Tim Mosmann, Richard H. Scheuermann, Elaine Reed, Karolina Palucka, Virginia Pascual, Bonnie B. Blomberg, Frank Nestle, Robert B. Nussenblatt, Ryan Remy Brinkman, Raphael Gottardo, Holden Maecker, J Philip McCoy

**Affiliations:** 1 Vaccine and Infectious Disease Division, Fred Hutchinson Cancer Research Center, Seattle, 98109, WA; 2 Hematology Branch, National Institutes of Health, Bethesda, Maryland, USA; 3 BD Biosciences, San Jose, CA, USA; 4 Terry Fox Laboratory , British Columbia Cancer Agency, V3J 4W6, Canada; 5 UCLA Pathology and Laboratory Medicine, Los Angeles, CA; 6 Dept of Neurology, Yale School of Medicine, New Haven, CT; 7 Baylor Institute for Immunology Research, Dallas, TX; 8 University of Cambridge, JDRF/Wellcome Trust Diabetes and Inflammation Laboratory, Cambridge Institute for Medical Research, Cambridge, UK; 9 Dept Microbiology & Immunology, University of Miami Miller School of Medicine, Miami, FL; 10 Guys and St Thomas Hospital, Guy’s Hospital, London, UK; 11 School of Pharmacy and Biomolecular Sciences, University of Brighton, Brighton, BN2 4GJ, United Kingdom; 12 Brighton and Sussex Medical School, Division of Medicine, Brighton, BN1 9PS, United Kingdom; 13 Department of Informatics, J. Craig Venter Institute, La Jolla, 92037, CA; 14 School of Mathematics and Physics, University of Queensland, Brisbane, Australia; 15 The Broad Institute of MIT and Harvard, Cambridge, MA 02142, USA; 16 Baxter Laboratory in Stem Cell Biology, Stanford University, Stanford, California, 94305, USA; 17 University of Rochester Medical Center, School of Medicine and Dentistry, Rochester, 14642, NY; 18 Laboratory of Immunology, National Eye Institute, National Institutes of Health, Bethesda, Maryland, USA; 19 Department of Medical Genetics, University of British Columbia, Canada; 20 Institute for Immunity, Transplantation, and Infection, Stanford University School of Medicine, Stanford, 94305, CA; 21 NHLBI Flow Cytometry Core, NIH, Bethesda, 20892, MD.

## Abstract

Standardization of immunophenotyping requires careful attention to reagents, sample handling, instrument setup, and data analysis, and is essential for successful cross-study and cross-center comparison of data. Experts developed five standardized, eight-color panels for identification of major immune cell subsets in peripheral blood. These were produced as pre-configured, lyophilized, reagents in 96-well plates. We present the results of a coordinated analysis of samples across nine laboratories using these panels with standardized operating procedures (SOPs). Manual gating was performed by each site and by a central site. Automated gating algorithms were developed and tested by the FlowCAP consortium. Centralized manual gating can reduce cross-center variability, and we sought to determine whether automated methods could streamline and standardize the analysis. Within-site variability was low in all experiments, but cross-site variability was lower when central analysis was performed in comparison with site-specific analysis. It was also lower for clearly defined cell subsets than those based on dim markers and for rare populations. Automated gating was able to match the performance of central manual analysis for all tested panels, exhibiting little to no bias and comparable variability. Standardized staining, data collection, and automated gating can increase power, reduce variability, and streamline analysis for immunophenotyping.

Flow cytometry is one of the most powerful tools for single-cell analysis of the immune system at a cellular level; yet it suffers from a lack of standardization beyond the simplest clinical assays that count major subsets. In research settings, each study tends to use its own combination of markers and fluorochromes, even when purportedly analyzing similar cell subsets. Sample handling, instrument type and setup, gating and analysis strategies, and ways in which the data are reported can all vary^1^,^2^. Unfortunately, these differences can all affect the results and how they are interpreted^3^,^4^,^5^,^6^,^7^,^8^,^9^.

The Human Immune Phenotyping Consortium (HIPC) was developed by the Federation of Clinical Immunology Societies (FOCIS) to address these issues by promoting standardization of flow cytometry immunophenotyping in clinical studies, so that data could be compared across sites and studies. As part of these efforts, the HIPC immunophenotyping panel was developed^2^. The HIPC panels consist of five eight-color antibody cocktails, designed to phenotype major immune cell subsets in peripheral blood mononuclear cells (T cells, Treg, Th1/2/17, B cells, and NK/dendritic cells/monocytes). These panels were designed to standardize routine immunophenotyping in humans while still being compatible with widely available clinical flow cytometers. Although they were not designed to represent the full complexity of cutting-edge research, the cocktails were designed to be easily expanded with additional colors to serve that purpose. The Euroflow consortium^7^,^10^,^11^,^12^ and the ONE Study^13^ have successfully developed standardized immunophenotyping panels and procedures for Leukemia and Lymphoma diagnostics and whole blood immunophenotyping, respectively^13^.

Here we demonstrate that an automated data analysis strategy can be integrated into a workflow utilizing a standardized staining panel.

Following development and testing of the HIPC panels, lyophilized reagent cocktails in 96-well plates were developed (BD Lyoplate, BD Biosciences, San Diego, CA). The use of lyophilized reagent cocktails is a proven method for improving standardization^3^,^14^,^15^, in that it protects against errors of reagent addition or mis-titration, provides improved reagent stability, and simplifies assay setup.

In addition to antibodies and reagent differences, analysis strategies for flow cytometry data remain highly non-standardized making results difficult to reproduce and compare across experiments. Traditionally, the majority of flow cytometry experiments have been analyzed visually, either by serial manual inspection of one or two dimensions at a time (a process termed “gating”, with boundaries or “gates” defining cell populations of interest). However, these visual approaches are labor intensive and highly subjective, and they neglect information present in the data that are not visible to the human eye, thus representing a major obstacle to the automation and reproducibility of research. For example, in a study of Intracellular Cytokine Staining (ICS) standardization involving 15 institutions, the mean inter-laboratory coefficient of variation ranged from 17 to 44%, even though the cell preparation was standardized and the testing was performed by using the same samples and reagents at each site^3^. Most of the variation observed was attributed to gating, even though experts in the field had conducted the analyses. It was concluded that the analysis, particularly gating, was a significant source of variability, and it was suggested that analysis strategies should be standardized.

Over the past eight years, there has been a surge in the development and application of computational methods for flow cytometry data analysis in an effort to overcome limitations in manual analysis^16^ and the importance of automated, high-dimensional analysis was highlighted in a recent position paper^17^. Pedreira *et al.* showed significant correlation between automated gating and manual data analysis of PBMC subsets and could discriminate between normal and reactive samples and B-cell chronic lymphoproliferative disorders^18^,^19^. Fiser *et al.* showed how hierarchical clustering with a Mahalanobis distance metric could be used to classify PBMCs into different phenotypic subsets with good agreement to manual analysis^20^. Although their approach was limited to relatively small numbers of events due to computational limitations, it demonstrated the utility of an unsupervised approach that takes into account the information in the full multidimensional data. Recently, the FlowCAP (Flow Cytometry: Critical Assessment of Population Identification Methods) consortium provided an objective approach to compare computational methods with both manual gating and external variables using statistical performance measures^21^. Based on the results of these study, Aghaeepour *et al.* concluded that computational methods had reached a sufficient level of maturity and accuracy for reliable use in flow cytometry data analysis.

Based on these encouraging results, we hypothesized that computational algorithms could be used to improve the standardization of flow cytometry results beyond what can be accomplished by the standardization of the wet lab component alone. In order to select the best computational methods for this task, we leveraged the FlowCAP project to compare and select the best performing algorithms based on a pilot dataset. The best-performing algorithms were combined using the OpenCyto framework^22^ to leverage the best features of each, and compared to a central manual analysis in terms of variability and bias on four staining panels using both lyophilized and cryopreserved control cells.

## Materials and Methods

### Cells

Lyophilized control PBMC (CytoTrol, Beckman Coulter, Miami, FL) were reconstituted and used according to the vendor’s instructions. Cryopreserved PBMC from three donors were frozen in replicate vials of 10^7^ cells per vial and obtained from Precision R&D (Frederick, MD).

### Staining cocktails and lyophilized reagent plates

The HIPC Immunophenotyping panels are listed in Table 1. These staining panels were designed to identify the major subsets of B cells, T cells, T-helper cells, and dendritic cells, monocytes, and NK cells^2^. All reagents were first tested and optimal titers determined among three of the nine participating HIPC laboratories.

The lyophilized reagent plates, along with a consensus staining protocol, were distributed to nine international laboratories for cross-site testing. The protocol included fluorescence target channels for use with pre-stained single-color control beads included in the reagent plates. Two experiments were performed: one with lyophilized control cells (CytoTrol, Beckman Coulter, Miami, FL), and the other with replicate vials of cryopreserved PBMC from three healthy subjects (Precision R&D). Data collected included manual analysis for the specified cell subsets, as performed at each site, FCS files, from which central analyses (manual and automated) were performed, and instrument setup parameters. The cell subsets pre-specified for evaluation in the study are shown in Table 2. The number of cell events per FCS file for each staining panel varied widely across centers. They are summarized here by their median (min, max): T-cell: 125,900 (44,680, 483,300), DC/Mono/NK: 108,300 (49,330, 474,600), B-cell: 141,600 (38,900, 449,200), T-regulatory: 135,500 (54,340, 458,400).

### Design of inter-laboratory experiments

In the initial cross-site experiment, nine sites stained four replicates of lyophilized control cells (CytoTrol) in lyophilized reagent plates. Lyophilized control cells were chosen in order to eliminate variability, as cryopreservation and thawing of PBMC were expected to introduce considerable staining variability. However, lyophilization was also found to alter the staining profile of certain markers, compromising the assessment of some populations (e.g., those involving IgD). All sites used either a Fortessa or LSR cytometer (Becton Dickinson, San Jose, CA).

In the second cross-site experiment, nine sites stained three replicates of each of three cryopreserved PBMC samples, to assess variability in the context of real-life samples (cryopreserved and thawed PBMC). The same lot of lyophilized reagent plates was used for both experiments. Sites provided results of gating for defined cell subsets using their own gating schema based on general instructions for the experiment from lyophilized samples (provided in Supplementary Material), as well as FCS files for a centralized analysis from both experiments. Only eight sites returned data for the second experiment, and one of the eight was excluded since they did not collect one of the required markers/channels in each of the panels.

### Central manual analysis

FCS files submitted by each participating site consisted of triplicates of each of three samples stained with the five cocktails included in the lyoplate.

These were accompanied by FCS files of the single-stained compensation bead samples in the lyoplate, To optimize compensation for centralized analysis, post-acquisition data analyses were performed using FlowJo (Tree Star Inc., version 9.6.3). Tube-specific matrices were constructed for each site, necessitated by the tandem (APC-H7, PE-Cy7) conjugates associated with each of the five cocktails.

Initial filtering of data from each cocktail delineated lymphoid or mononuclear populations using FSC-A/SSC-A profiles and excluded doublets using FSC-A/FSC-H profiles and dead cells using FSC-A/fixable green live-dead profiles. Subsequent gating was designed to identify major lymphocyte and monocyte cell populations specified previously (2). The design of the lyoplate did not offer the opportunity to establish gate placement using Fluorescence-Minus-One (FMO) controls. Therefore, guidance for gate placement was accomplished by setting up FMO controls using the same liquid reagents that were used in lyophilized form in the lyoplate.

In two instances (B-cell, Treg) Boolean gates were constructed to aid in identifying several populations. Gating schemes for all panels can be found in Supplementary Figures 1–4, and live visualization of the manual (and automated) gates for each sample can be found online at the ImmuneSpace portal.

The *flowWorkspace* (v 3.15.17) package^20^ was used to import the manually gated data into R for further analysis^19^. Manual gate import scripts can be found online at ImmuneSpace^23^. Of the nine centers, one center failed to submit results, and one center was excluded from the analysis because markers in the FCS file were mislabeled and could not be matched to the expected panels.

### Automated analysis algorithms

The two top performing gating algorithms *-* OpenCyto (v. 1.7.4)^22^, flowDensity (v. 1.4.0)^24^ - in a study run by the FlowCAP consortium aimed at selecting the best performing algorithms for this larger study were chosen for the analysis presented in this paper. (See Supplementary Figures 5–6). Gating was performed using OpenCyto plug-in algorithms^22^,^24^, enabling different gating algorithms to be selected for different steps of the gating pipeline for each panel, depending on their strengths.

OpenCyto is a BioConductor framework for constructing robust and reproducible end-to-end flow data analysis pipelines. The framework can handle large data sets in a memory efficient manner and allows the incorporation of domain-specific knowledge by encoding hierarchical relationships between cell populations as part of the pipeline, making it ideal for reproducing hierarchical manual analysis. Pipeline templates are defined through a text-based csv file, promoting reusability and eliminating the need to write data-set specific code. OpenCyto supports several general purpose data-driven gating approaches natively, as well as user-defined methods via a plug-in framework.

flowDensity is based on a supervised sequential bi-variate clustering approach that generates a set of pre-defined cell populations. It chooses the best cut-off for individual markers using characteristics of the density distribution and takes just seconds to run per file. flowDensity is available as R/BioConductor package, and is integrated into OpenCyto as a plug-in.

The Thelper panel was excluded in preliminary analysis as too variable to be usable. OpenCyto gating templates for four of the lyoplate panels (B-cell, T-cell, T-regulatory, and DC/Mono/NK)), as well as R code used to perform the automated gating, import manually gated data, data cleaning, statistical analysis and plotting are available through the ImmuneSpace portal (https://www.immunespace.org/project/HIPC/Lyoplate/begin.view). The gating of the FCS files from templates takes approximately 45 minutes, although setting up the initial templates is an iterative process requiring substantially more time. Automated and manual gates can be visualized across samples and centers through an interactive web application built within the ImmuneSpace database. In addition, all analyses performed here are provided as R reports that can be rerun by any users on the web-server providing complete transparency of code and results including gating and statistical modeling of gated population statistics. Raw and processed data can also be easily downloaded, which can be used to reproduce the analyses locally or perform novel analyses. Automated and manual gate definition can [Fig f1][Fig f2]also be exported in Gating-ML^25^, an open standard extensible markup language for describing flow cytometry gating, as well as CLR format .

### Statistical Analysis

Cell population statistics were extracted from the manual and automated gating [Fig f3]approaches using R’s flow cytometry tools and analyzed as described below.

Different sources of variability (center, sample, and residual) were assessed by fitting a linear mixed effects model to the proportion of cells identified in each cell population in each staining panel. For a fixed staining panel, cell subset, and gating method, we let *p*_*rij*_ represent the proportion of cells in replicate *r* from sample *i*, and center *j*. We transformed the proportion, (1): *y*_*rij=*_*logit*(*p*_*rij*_), and model (2):


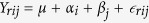


where *μ*_*i*_ are the intercepts, *α*_*i*_ are the sample-level random effects, *β*_*j*_ are the center-level random effects, and *∈*_*rij*_ are the residual technical errors, with 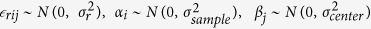. The estimates of the 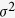 ’s from the model are the components of variance due to the different sources of variability (Fig. 4). Sample-level estimates were obtained by replacing the sample-level random effects, *α*_*i*_ , in the above model, with fixed effects for which we obtained confidence intervals in Fig. 5.

We defined bias as the difference between sample-level estimates of population proportions for automated gating and sample-level estimates of population proportions for manual gating, after adjusting for center-to-center variability, and taking into account the 95% confidence intervals on those estimates. For a given population and sample *i* , bias is defined as 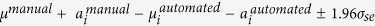.

## Results

### Individual versus central manual analysis

**Figure 1 f1:**
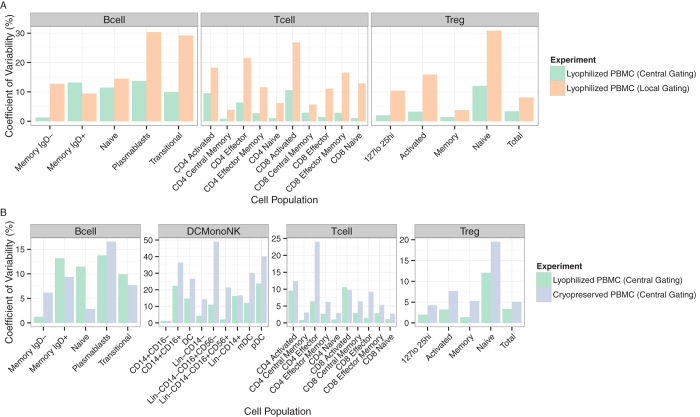
Individual and central manual analysis of B-cell, T-reg, T-cell subsets. (**A**) CV’s between sites are shown for each subset from the lyophilized cell (Cyto-trol) experiments. Centralized gating decreases the coefficient of variability for nearly all cell populations (Memory IgD+ cells in the B-cell panel are an exception) across all staining panels. Site-specific gating strategies for the DC/Mono/NK panel were non-comparable (no CVs shown). (**B**) Comparison of inter-site CVs for cryopreserved and lyophilized cells. CVs for cryopreserved cells are generally larger than for lyophilized cells (with the exception of IgD+ cell populations in the B-cell panel). For the lyophilized cell protocol, 68 files were analyzed and for the cryopreserved cell protocol, 60 files were analyzed for each panel.

Central manual analysis significantly reduced the variability in comparison with individual site analysis (Fig. 1A). This was not unexpected based on previous studies^3^, and given that the individual site analysis was done without a shared gating template and with only general instructions as to how each particular cell subset (e.g., CD3+CD4+ lymphocytes) was to be gated^2^. We further compared the two experiments using only the data from central manual analysis (Fig. 1B). In general, except for those subsets that could not be effectively identified using lyophilized cells, the CV’s were similar or slightly lower for the lyophilized cells compared to the cryopreserved PBMC (Fig. 1B).

### Findings from central manual analysis

**Figure 2 f2:**
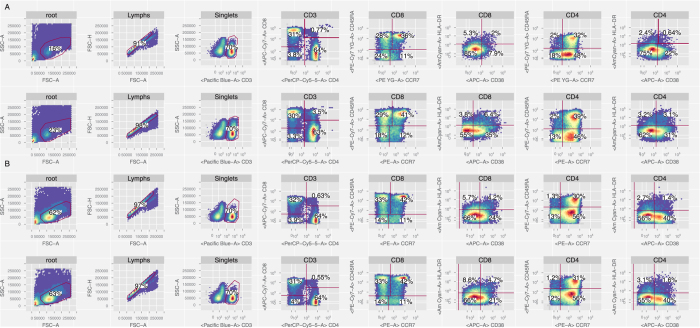
Example of inter- and intra-site variability from experiment 1 (lyophilized cells). (**A**) Examples of T-cell panel gating from two sites. (2 files analyzed) (**B**) Two replicates of the T cell panel from one site. (2 files analyzed).

The within-site replicates for both experiments were very good, for essentially all cell subsets. In general, consistency between sites was more variable than within sites (representative examples of inter and intra-site variability from the T-cell panel are shown in Fig. 2A,B, respectively). The within-site coefficients of variability for the different cell populations and panels were reduced by between 94% and 43% (mean 73%, IQR 18%) compared to the between-site CVs for the same panels and populations. While larger, more easily identified subsets (e.g., CD3+ and CD4+ T cells) tended to have CV’s of <10% across sites, subsets that were difficult to identify due to dim staining, and/or that required multiple successive gates, had higher CV’s. While these results are not surprising, they do highlight the challenges of cross-site flow cytometry data analysis and the need for more standardized and objective data analysis approaches.

### Automated analysis of cryopreserved PBMCs reduces technical (center-to-center) variability for some subsets

**Figure 3 f3:**
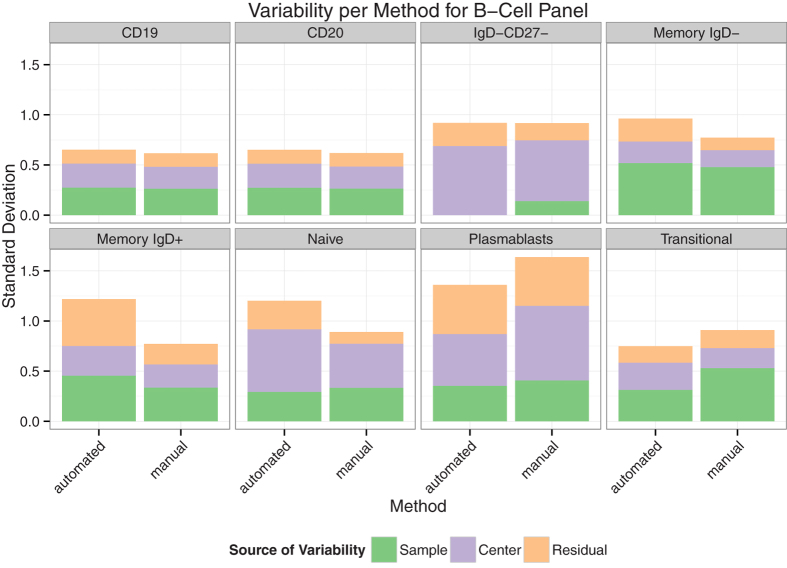
Center, biological and residual variability per population and gating method for the B-cell panel. For most cell populations, the center and sample variability were comparable across automated and manual gating methods. The IgD marker is poorly resolved as evidenced by the higher variability in automated analysis. Y-axis is the standard deviation of the center, sample and residual components estimated from the random effects model. (n = 63 files).

We assessed which experimental factors had the largest impact on the variability of estimated population statistics from the three gating methods using a linear mixed model. In the T, B and T-regulatory panels, the majority of measured cell populations exhibited biological variation that was larger than technical variation. In contrast, for the DC panel, technical variability was the primary source of variation in the data for the majority of measured cell populations (Fig. 3, and Supplementary Figures 7–9). The residual variation captures variability due to other sources not explicitly captured by the model. We examined the performance of individual panels more closely.

In the T-cell and B-cell panels, the OpenCyto and flowDensity methods generated cell population estimates with lower variability compared to manual gating for some cell populations. Specifically the transitional B-cell and plasmablast populations, and the CD4 effector, CD4 effector memory, CD8 central memory and CD8 effector populations were improved (Fig. 3 and Supplementary Figure 7).

The CD8 activated and effector memory cell subsets were problematic for automated methods, as seen in scatterplots of manual vs. automated cell population estimates (Supplementary Figure 10). Both cell populations exhibited poor concordance between automated and manual gating across multiple centers, and larger total variation than manual gating. Likewise in the B-cell panel, naive and IgD-containing cell subsets (memory IgD+ and IgD−) had larger total variability for automated versus manual gating and poor concordance across centers for low abundance (low proportion) cell subsets.

### Automated algorithms recapitulate manual analyses with low bias

**Figure 4:**
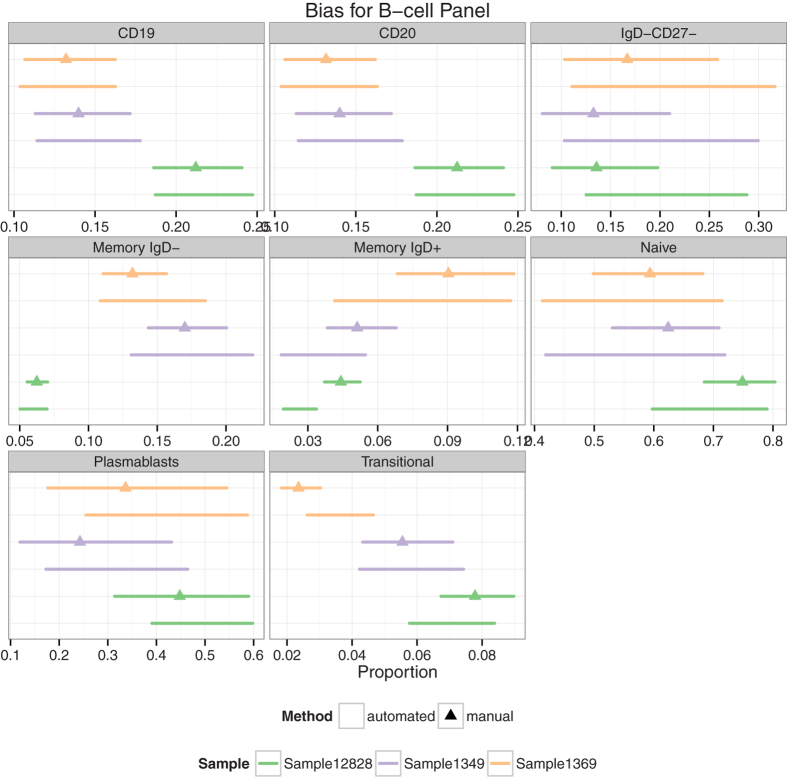
Estimated cell proportions from each population and gating method in the B-cell panel. Estimated proportions and 95% confidence intervals are shown for each sample, gating method, and cell population in the B-cell panel. There is little bias in automated gating compared to central manual gating, with the exception of small differences in automated gating for rare populations based on poorly resolved markers such as Memory IgD+. Data for other panels is shown in the Supplementary Material. (n = 63 files).

In addition to variability as a metric of performance, we are also interested in evaluating the bias (i.e. whether the point estimates differ significantly between manual and automated gating). Figure 4 shows population proportion estimates and 95% confidence intervals for each subset, method, and donor in the cryopreserved PBMC B-cell panel. In general, the estimates are comparable manual and automated gating as evidenced by the overlapping confidence intervals across methods, indicating that any differences between point estimates are not significant.

Cell subsets that did show differences were investigated further (memory IgD+/− and transitional B cells). The increased variability in the cell populations defined by the IgD can be explained by the poor resolution of IgD in some centers where there is little information in the data to delineate positive and negative cells (Supplementary Figure 11). An example is center G, where the naive and IgD-CD27- cell population estimates are outliers compared to the other centers (Supplementary Figures 12, 13). In other instances the upstream manual gating could be identified as sub-optimal in some samples, impacting downstream population estimates (e.g., Plasmablasts, Supplementary Figure 14).

Low abundance cell populations were not always problematic for automated gating. In the T-regulatory cell panel, automated gating performed surprisingly well relative to central manual gating (Supplementary Figures 15,16). The cell population estimates showed little to no bias (Supplementary Figure 17). While the T-regulatory cell populations were amongst the cell subsets with the lowest proportions considered, automated methods performed well, indicating that the success of automated gating depends on the ability of a panel to resolve cell subpopulations, perhaps more so than the prevalence of the cell subsets within the panel.

Despite large technical variability, the DC/Mono/NK panel was entirely consistent with manual gating and the population estimates were relatively unbiased (Supplementary Figures 18,19 and 20). Unfortunately, the substantial technical variation overwhelmed the biological variation, rendering the panel impractical for detecting changes in cell frequency due to biological effects.

The T-cell panel performance was consistent with the T-regulatory and B-cell panels. Most cell population estimates were comparable to manual gating with little bias (Supplementary Figures 21, 22). Problematic populations included the CD8 activated cell subset, which was based on poorly resolved markers and had low abundance, making it difficult to identify in a data-driven manner (Supplementary Figure 10), as well as CD8 effector and CD4 effector memory T cells. These cell populations showed some bias compared to manual gates, but examination of the automated gates demonstrated that their placement was, nonetheless, reasonable and the observed bias is due to an accumulation of subtle differences in the upstream gating.

### Reagent and analysis standardization won’t replace good laboratory practices

By examining the cell population statistics from centralized manual gating and comparing them to automated analysis, we identified centers that were outliers for certain cell populations in certain staining panels. One such example is the previously mentioned B-cell subsets in center G.

A number of other centers had outlier populations in the T-cell panel, including Center F for CD4 effector cells from sample 12828, Center B for CD8 effector memory cells from sample 1369, and Center C for CD8 naive cells from sample 12828. Closer examination of pairwise event plots for the relevant samples from these centers identified data quality issues possibly related to protocol adherence.

Center B failed to collect the scatter channels that allowed for gating of singlets for the T-cell panel, thus none of the samples from Center B had a singlet gate for the centralized manual gating scheme (Supplementary Figure 23). Inspection of the dot plots did not immediately reveal the cause of the difference, but samples from Center B did exhibit poor resolution in the CCR7 dimension (Supplementary Figure 24). Samples from Center C appeared to have problematic compensation in the CD45RA and CD197 (CCR7) dimensions (Supplementary Figure 25), leading to drastically different cell population distributions for the CD8 effector / memory T-cell subsets from other centers. One of the replicates from Center B sample 12828 exhibited a trimodal CD3 distribution (Supplementary Figure 26), accounting for the outlier nature of this sample. While standardization of reagents (via lyoplates) and harmonization of analysis pipelines can sometimes address data quality issues caused by differences in protocol adherence between centers or inadequate quality control and compensation issues), such problems are still best addressed through detailed SOPs, quality control, and proficiency testing.

### Power analysis indicates centralized gating can help control for technical effects

**Figure 5:**
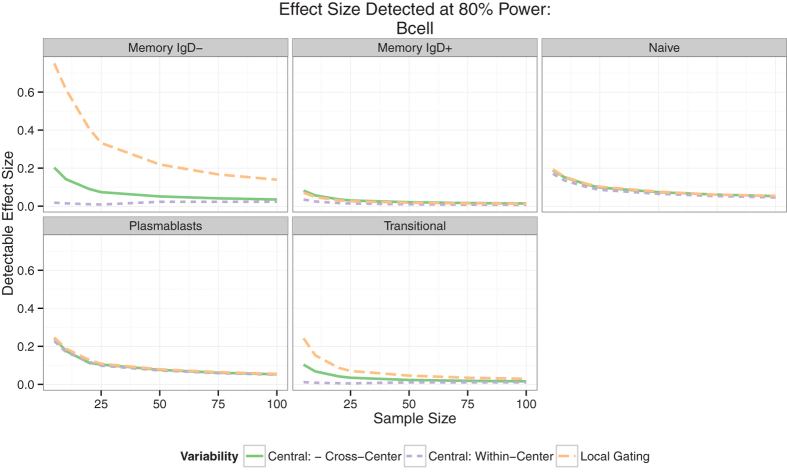
Power analysis comparing site-specific and central gating for the B-cell panel. Assuming 80% power and a 5% significance level, the expected minimum detectable effect size is shown for each cell population as a function of increasing sample size. The change in sensitivity is shown for site-specific gating (dotted line), central gating in the absence (dashed line) of and in the presence (solid line) of center-to-center variability. Site-specific vs. standardized central gating has a larger impact on the sensitivity of the assay than center-to-center technical variability. Data for other panels is shown in the supplementary material.

In order to assess the relative importance of different sources of variability and their impact on statistical power, we performed a power analysis for each staining panel. We calculated the minimum detectable effect size at 80% power (the probability of detecting a difference in the cell population proportion due to treatment if one truly exists) for varying sample sizes with different assumptions about the observed sources of variability (i.e., assuming the data are gated locally, gated centrally, or data are generated from a single center). We evaluated the minimum effect size for each cell subset in each panel, using an average estimate of the variability from the different centralized (manual and automated) gating approaches, rather than any specific gating method. Variance estimates due to technical effects (center-to-center), biological effects (sample-to-sample) and differences in local vs. central gating,were drawn from mixed effects model fits. Our results demonstrate that most of the benefit in increased power comes from centralized and standardized gating (Fig. 5 and Supplementary Figures 27,28), and that additional benefits from eliminating center-specific effects are relatively minor, but can be moderated by increasing sample size. These results also show which cell subsets in each panel exhibit significant technical variability.

## Discussion

The HIPC created a set of lyophilized standard 8-color immunophenotyping cocktails that allow for standardized cell subsetting. Since the data were acquired on high-end instruments, differences in laser power and filters can contribute to site-to-site variability. A standard protocol for use of these plates was developed. Together with detailed target values for setting PMT voltages, we hypothesized that this approach would provide the ability for highly reproducible immunophenotyping across sites. While this was achieved for most basic cell subsets, it is clear that optimal reagent and instrument performance is needed for consistent results with minor and “dim” subsets. It is not entirely clear in advance which fluorochromes/antibodies will work well in a dried down cocktail. In case of IgD the liquid format was not giving optimal resolution and consequently the staining of the lyophilized reagent was also poor. The poor resolution of IgD has a trigger effect on all its children populations. Replacement of this reagent with one that yields more distinct staining would improve reproducibility. Additional checks on instrument performance and adherence to staining and acquisition protocols would also likely increase the reproducibility for these more difficult to analyze cell subsets. More detailed gating instructions to centers could help reduce the impact of local gating on reproducibility, but would likely not achieve the precision of central gating since one analysts would have to observe samples gated at other centers.

Our analysis of these multi-site data indicates that central analysis is more reproducible than individual site analysis, as evidenced by significantly lower coefficients of variability (Fig. 1), and that automated algorithms can reproduce manual central analysis with comparable reproducibility and little to no bias. In most cases (e.g. B-cell and T-cell panels), automated analysis provided matched or lowered variability compared to manual analysis (e.g. plasmablasts, transitional B-cells, CD8 central memory, CD8 effector, CD4 effector memory), demonstrating that automated analysis can improve upon existing manual methods.

When manual and automated methods showed significant disagreement, this appeared to be associated with rare cell subsets (e.g. CD8 activated cells in the T-cell panel), or poorly resolved populations (e.g. IgD+ cells in the B-cell panel). In some cases, variability decrease appeared to be due to improved performance of the automated gating approach. For example, in the B-cell panel plasmablast cell subset, visual inspection of the event-level data showed that the automated gates for the upstream CD20- parent populations were more reasonable than the manual ones (Supplementary Figure 14). In other cases, disagreement could be traced back to centers not adhering to experimental protocols, resulting in problematic data quality (e.g. problems with compensation, or potential problems with staining or marker resolution. Such issues can sometimes be resolved by automated analysis, but highlight the important role of careful adherence to experimental protocol, quality control, detailed SOPs, and proficiency testing in cross-center studies.

The bias observed in some cell subsets in automated vs. manual gating could be traced back to an accumulation of subtle differences in the upstream gating. Importantly, none of these upstream gates were problematic. This raises an important issue with hierarchical gating. The dependencies between cell population definitions enable differences in upstream gates to propagate through to downstream populations. Data-driven automated gating can mitigate this issue through consistency and reproducibility.

Using the standardized lyoplates combined with a unified gating strategy utilizing automated methods it was possible to resolve biological variation between samples for the T-cell, B-cell, and T-regulatory panels, while the technical variability in the DC/Mono/NK panel was too large to reliably resolve biological differences between samples. Particular care is needed if utilizing this panel in a cross-center setting. It is important to note that the automated gating strategy proposed for these standardized panels could likely be replaced by an alternate gating strategy to define the same cell populations with comparable results. We stress that the important factor for success is consistency in the gating strategy and consistency in the application of experimental protocols. While considerable effort is required to perform a centralized manual analysis of large cross-center data sets, this work shows that manual analysis efforts can be reduced as automated gating analysis can be applied with confidence using the methods profiled here.

In addition to being automated, and thus less time-consuming, computational methods lead to analyses that are objective, reproducible, and reusable across data sets that utilize common staining panels. These tools coupled with standardized experimental standard operating procedures should make it possible to more easily compare and integrate data across multiple sites, which will open the door to novel cross-center studies that would not be possible otherwise.

This study follows the “open science” trend by providing complete transparency of data and results, ensuring that reproducibility can be verified^21^,^22^,^26^. All materials, including primary data files, processed data, workspaces and analysis code, are made freely available using existing data standards and providing a valuable resource to the experimental and computational communities.

## Additional Information

**How to cite this article**: Finak, G. *et al.* Standardizing Flow Cytometry Immunophenotyping Analysis from the Human ImmunoPhenotyping Consortium. *Sci. Rep.* **6**, 20686; doi: 10.1038/srep20686 (2016).

## Supplementary Material

Supplementary Information

## Figures and Tables

**Table 1:** The HIPC antibody panel, specificities and clones.

	T cell	Treg	B cell	DC/mono/NK	Th1/2/17
FITC	dead	dead	dead	dead	dead
PE	CCR7 (150503)	CD25 (2A3)	CD24 (ML5)	CD56 (B159)	CXCR3 (1C6/CXCR3)
PerCP-Cy5.5	CD4 (SK3)	CD4 (SK3)	CD19 (SJ25C1)	CD123 (7G3)	CD4 (SK3)
PE-Cy7	CD45RA (L48)	CCR4 (1G1)	CD27 (M-T271)	CD11c (B-LY6)	CCR6 (11A9)
APC	CD38 (HIT2)	CD127 (HIL-7R-M21)	CD38 (HIT2)	CD16 (B73.1)	CD38 (HIT2)
APC-H7	CD8 (SK1)	CD45RO (UCHL1)	CD20 (2H7)	CD3+19+20 (SK7, SJ25C1, 2H7)	CD8 (SK1)
V450	CD3 (UCHT1)	CD3 (UCHT1)	CD3 (UCHT1)	CD14 (MPHIP9)	CD3 (UCHT1)
V500	HLA-DR (G46-6)	HLA-DR (G46-6)	IgD (IA6-2)	HLA-DR (G46-6)	HLA-DR (G46-6)

**Table 2:** Cell populations evaluated by the HIPC panels.

Panel	Population Name	Reliability	Corresponding Markers
T-cell	CD8 Activated	−	CD3+/CD8+/CD4− /CD38+/HLADR+
T-cell	CD4 Activated	+	CD3+/CD8−/CD4+/CD38+/HLADR+
T-cell	CD4 Central Memory	−	CD3+/CD8−/CD4+/CCR7+/CD45RA−
T-cell	CD8 Central Memory	−	CD3+/CD8+/CD4−/CCR7+/CD45RA−
T-cell	CD4 Effector	+	CD3+/CD8−/CD4+/CCR7−/CD45RA+
T-cell	CD8 Effector	+	CD3+/CD8+/CD4−/CCR7−/CD45RA+
T-cell	CD4 Effector Memory	+	CD3+/CD8−/CD4+/CCR7−/CD45RA−
T-cell	CD8 Effector Memory	−	CD3+/CD8+/CD4−/CCR7−/CD45RA−
T-cell	CD4 Naïve	+	CD3+/CD8−/CD4+/CCR7+/CD45RA+
	CD8 Naïve	+	CD3+/CD8+/CD4−/CCR7+/CD45RA+
B-cell	IgD−/CD27−	−	CD3−/CD19+/CD20+/IgD−/CD27−
B-cell	Transitional	+	CD3−/CD19+/CD20+
B-cell	Plasmablasts	−	CD3−/CD19+/CD20−/Cd24^high^/CD38^high^
B-cell	Naïve B	+	CD3−/CD19+/CD20+/CD27−/IgD+
B-cell	Memory IgD+	+	CD3−/CD19+/CD20+/IgD+/CD27+/IgD+
B-cell	CD19	+	CD3−/CD19+
B-cell	CD20	+	CD3−/CD20+
B-cell	Memory IgD-	+	CD3−/CD19+/CD20+/CD27+/IgD−
T-regulatory	Total T-regulatory	+	CD3+/CD4+/CD8-/LoCD127/HiCD25/CCR4+ (as % of CD4)
T-regulatory	Memory T-regulatory	+	CD3+/CD4+/CD8−/LoCD127/HiCD25/CCR4+/CD45RO+ (as % of total Treg)
T-regulatory	Naïve T-regulatory	+	CD3+/CD4+/CD8−/LoCD127/HiCD25/CCR4+/CD45RO− (as % of total Treg)
T-regulatory	CCR4-/CD45RO−	−	CD3+/CD4+/CD8−/LoCD127/HiCD25/CCR4−/CD45RO− (as % of parent)
T-regulatory	CCR4-CD45RO+	−	CD3+/CD4+/CD8−/LoCD127/HiCD25/CCR4−CD45RO+ (as % of parent)
T-regulatory	CCR4-HLADR−	+	CD3+/CD4+/CD8−/LoCD127/HiCD25/CCR4−HLADR− (as % of parent)
T-regulatory	CCR4-/HLADR+	−	CD3+/CD4+/CD8−/LoCD127/HiCD25/CCR4−/HLADR+ (as % of parent)
T-regulatory	CCR4+/CD45RO−	−	CD3+/CD4+/CD8−/LoCD127/HiCD25/CCR4+/CD45RO− (as % of parent)
T-regulatory	CCR4+/HLADR+	+	CD3+/CD4+/CD8−/LoCD127/HiCD25/CCR4+/HLADR+ (as % of parent)
T-regulatory	Total CD4	+	CD3+/CD4+/CD8− (as % of parent)
T-regulatory	LoCD127/HiCD25	+	CD3+/CD4+/CD8−/LoCD127/HiCD25 (as % of parent)
T-regulatory	Activated	+	CD3+/CD4+/CD8−/LoCD127/HiCD25/CCR4+/HLADR+ (as % of total Treg)
DC/Mono/NK	CD11c-/CD123-	−	CD11c−/CD123−
DC/Mono/NK	CD11c-/CD123+	+	CD11c−/CD123+
DC/Mono/NK	CD11c+/CD123−	+	CD11c+/CD123−
DC/Mono/NK	CD11c+/CD123+	–	CD11c+/CD123+
DC/Mono/NK	CD14+/CD16+	−	CD14+/CD16+
DC/Mono/NK	CD16-/CD56+	+	CD16−/CD56+
DC/Mono/NK	CD16+/CD56-	−	CD16+/CD56−
DC/Mono/NK	CD16+/CD56+	+	CD16+/CD56+
DC/Mono/NK	HLADR+	−	HLADR+
DC/Mono/NK	Lin-CD14−	+	Lin-CD14−
DC/Mono/NK	Lin−/CD14+	+	Lin−/CD14+
DC/Mono/NK	CD16−/CD56−	−	CD16−/CD56−

evaluated in the study, showing their common names and phenotypes (live, lymphocye, and singlet gates are not listed). Cell populations which could be reliably detected by automated gating in a panel are marked with a “+” in the “reliable” column, while those that were unreliable are marked with a “−”. We did not evalute the Th1/Th2/Th17 panel as it was determined early on in preliminary analysis that the panel was too variable to be reliable.
