# Supplementary material for: Standardizing Flow Cytometry Immunophenotyping Analysis from the Human ImmunoPhenotyping Consortium
Source: Sci Rep. 2016 Feb 10;6:20686. doi: 10.1038/srep20686 (PMC4748244; doi:10.1038/srep20686)
Supplement: Supplementary Information [file srep20686-s1.pdf]

# Supplementary Material for “Standardizing Flow Cytometry Immunophenotyping Analysis from the Human ImmunoPhenotyping Consortium”

Greg Finak[1,2,*], Marc Langweiler[3,*], Maria Jaimes[4], Mehrnoush Malek[2,5], Jafar Taghiyar[2,5], Yael Korin[6], Khadir Raddassi[7], Lesley Devine[7], Gerlinde Obermoser[8], Marcin L Pekalski[9], Nikolas Pontikos[9], Alain Diaz[10], Susanne Heck[11], Federica Villanova[11], Nadia Terrazzini[12], Florian Kern[13], Yu Qian[2,14], Rick Stanton[14], Kui Wang[2,15], Aaron Brandes[2,16], John Ramey[1,2], Nima Aghaeepour[2,5], Tim Mossman[2,18], Richard H Scheuermann[2,14], Elaine Reed[6], Karolina Palucka[8], Virginia Pascual[8], Bonnie B Blomberg[10], Frank Nestle[11], Robert B. Nussenblatt[19], Ryan Remy Brinkman[2,5,20,†], Raphael Gottardo[1,2,†], Holden Maecker[21,†] and J Philip McCoy Jr.[22,†]

[1]Vaccine and Infectious Disease Division, Fred Hutchinson Cancer Research Center, Seattle, WA, 98109

[*]Equal contribution

[2]FlowCAP Consortium

[3]Hematology Branch, National Institutes of Health, Bethesda, Maryland, USA

[4]BD Biosciences, San Jose, CA, USA

[5]Terry Fox Laboratory , British Columbia Cancer Agency, Canada, V3J 4W6

[6]UCLA Pathology and Laboratory Medicine, Los Angeles, CA

[7]Dept of Neurology, Yale School of Medicine, New Haven, CT
[8]Baylor Institute for Immunology Research, Dallas, TX
[9]University of Cambridge, JDRF/Wellcome Trust Diabetes and Inflammation Laboratory, Cambridge Institute for Medical Research, Cambridge, UK
[10]Dept Microbiology & Immunology, University of Miami Miller School of Medicine, Miami, FL
[11]Guys and St Thomas Hospital, Guy's Hospital, London, UK
[12]School of Pharmacy and Biomolecular Sciences, University of Brighton, Brighton, United Kingdom, BN2 4GJ
[13]Brighton and Sussex Medical School, Division of Medicine, Brighton, United Kingdom, BN1 9PS
[14]Department of Informatics, J. Craig Venter Institute, La Jolla, CA, 92037
[15]School of Mathematics and Physics, University of Queensland, Brisbane, Australia
[16]The Broad Institute of MIT and Harvard, Cambridge, MA 02142, USA.
[17]Baxter Laboratory in Stem Cell Biology, Stanford University, Stanford, California, USA, 94305
[18]University of Rochester Medical Center, School of Medicine and Dentistry, Rochester, NY 14642
[19]Laboratory of Immunology, National Eye Institute, National Institutes of Health, Bethesda, Maryland, USA
[20]Department of Medical Genetics, University of British Columbia, Canada
[21]Institute for Immunity, Transplantation, and Infection, Stanford University School of Medicine, Stanford, CA 94305
[22]NHLBI Flow Cytometry Core, NIH, Bethesda, MD 20892
[†]Equal contribution

October 18, 2015

# 1 Supplementary Figures

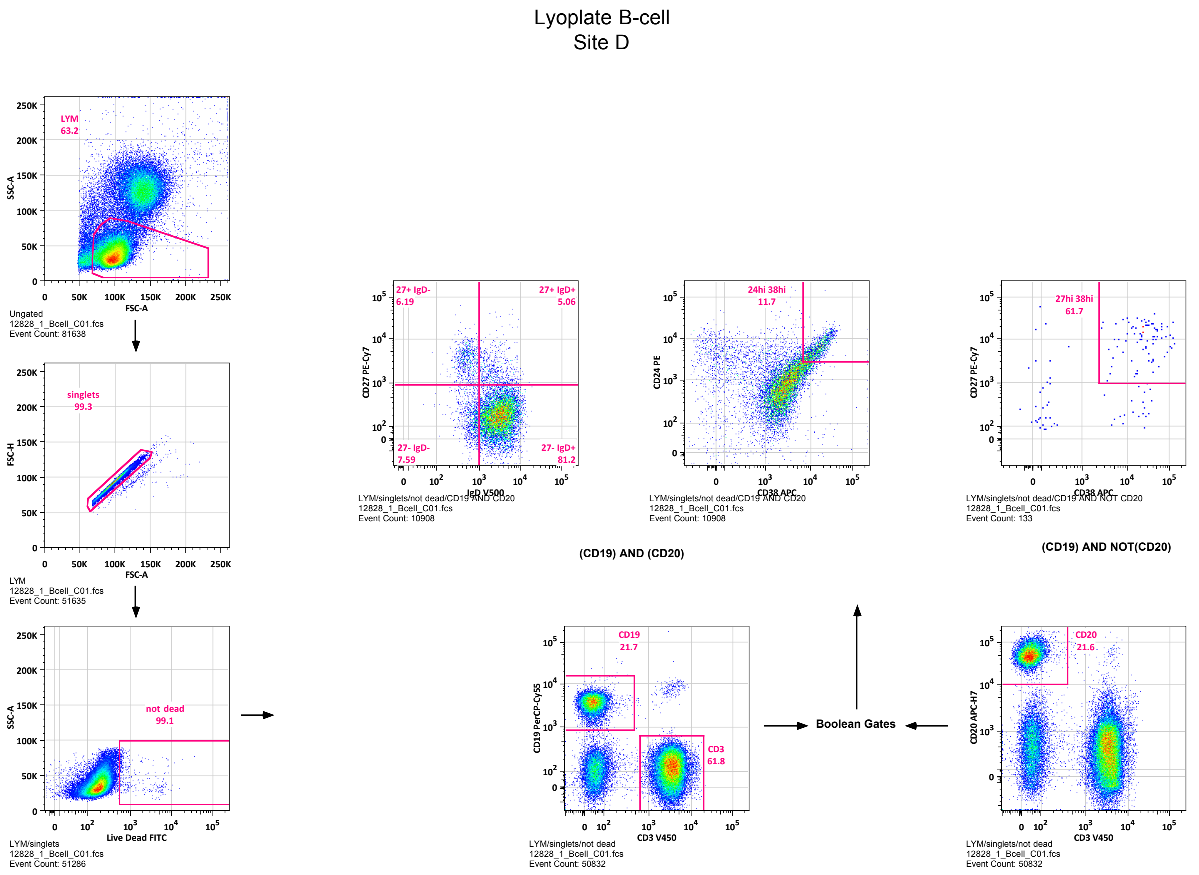


Supplementary Figure 1: An example centralized gating scheme for the B-cell panel from a sample run at site D.

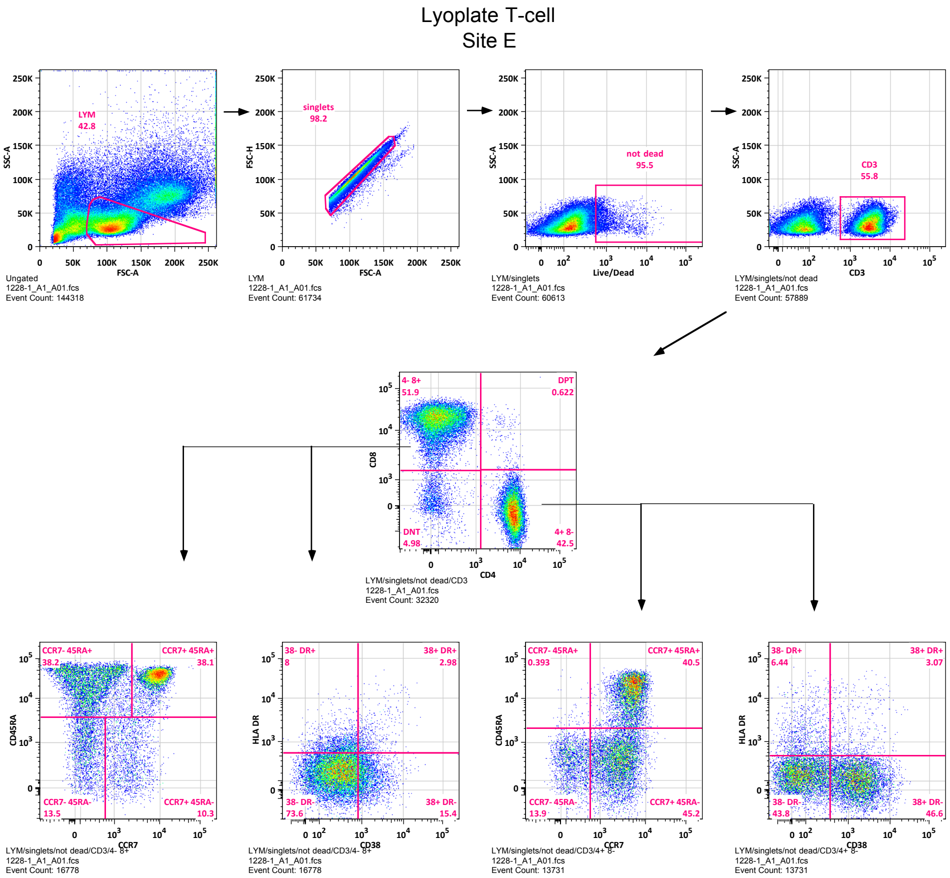


Supplementary Figure 2: An example centralized gating scheme for the T-cell panel from a sample run at site E.

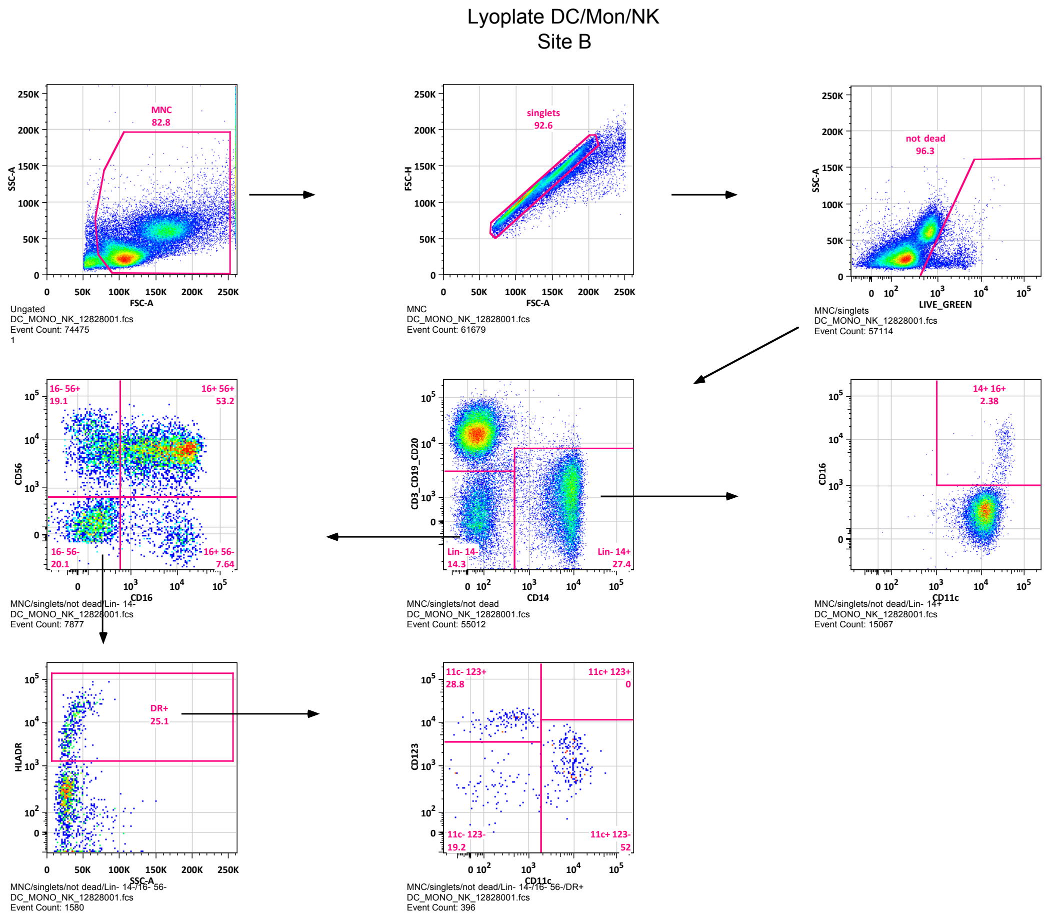


Supplementary Figure 3: An example centralized gating scheme for the DC/Mono/NK panel from a sample run at site B.

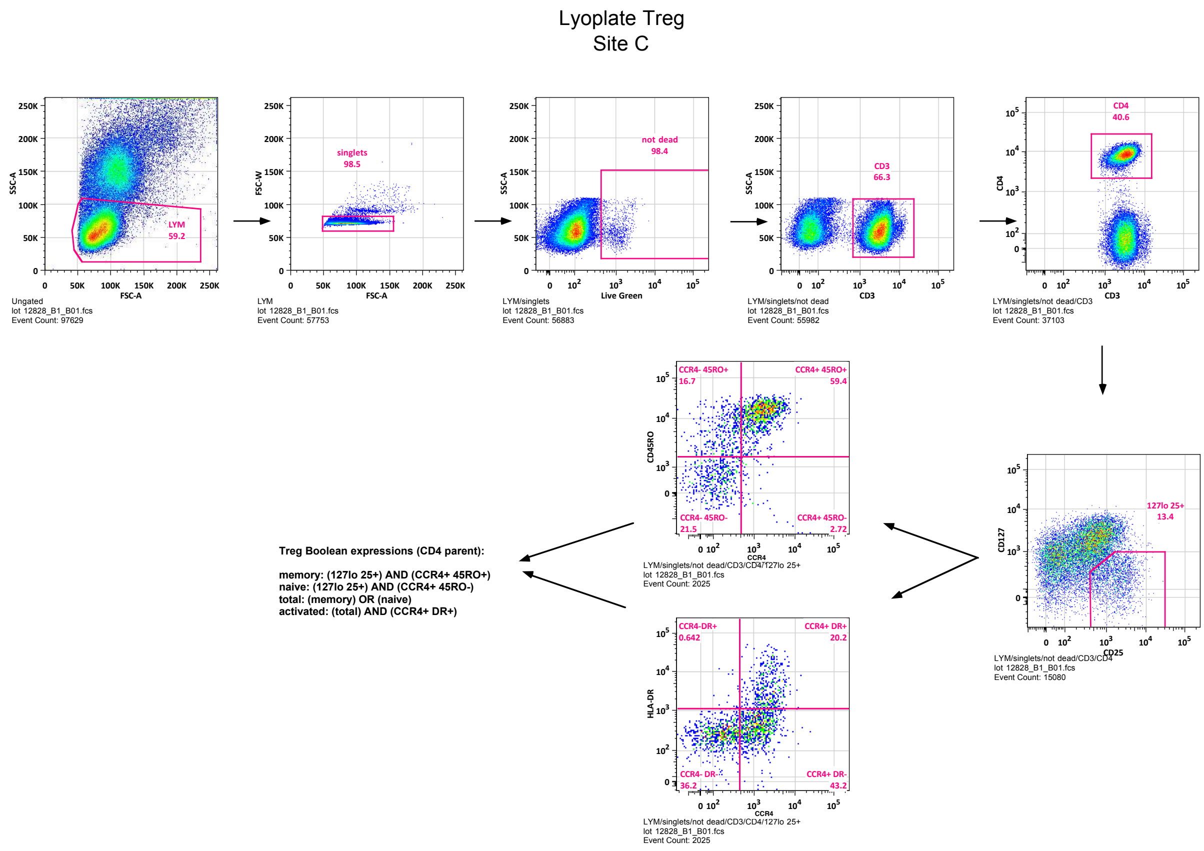


Supplementary Figure 4: An example centralized gating scheme for the T-reg panel from a sample run at site C.

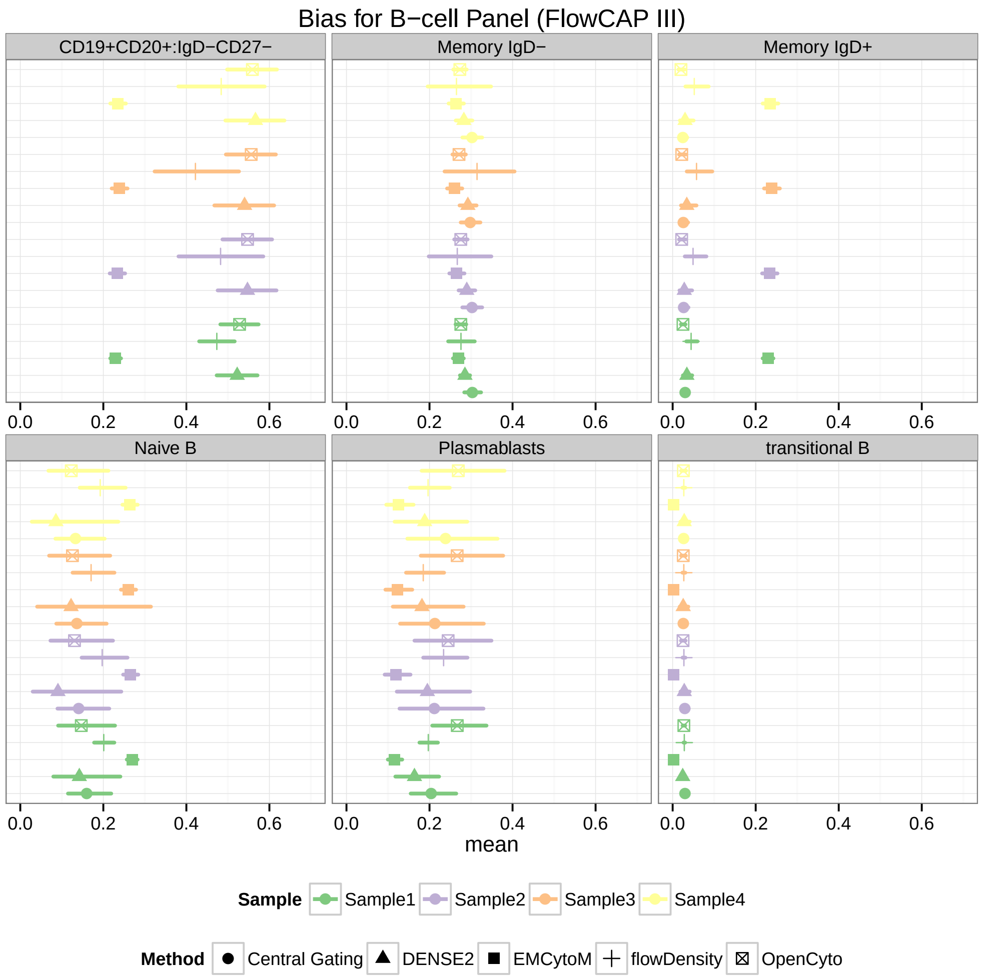


Supplementary Figure 5: Bias and variability for cell populations in the B-cell lyoplate panel from the lyophilized cell experiment from FlowCAP III. Colors indicate samples, shapes indicate gating methods. Most approaches were unbiased with respect to central manual gating for the B-cell panel and showed similar variability to central manual gating, with the exception of EMCytoM which exhibited biased cell populations estimates for Naive, Memory IgD+, transitional, and plasmablast cell subsets.

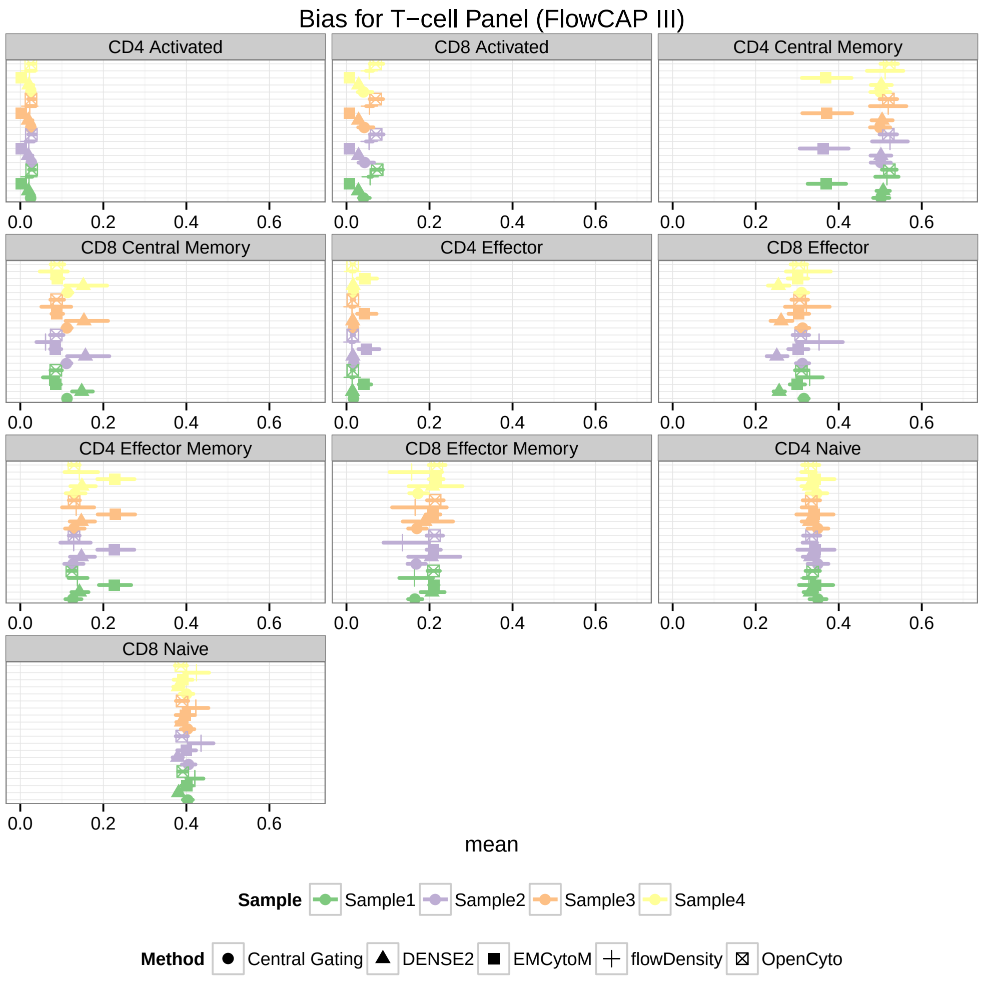


Supplementary Figure 6: Bias and variability for cell populations in the T-cell lyoplate panel from the lyophilized cell experiment from FlowCAP III. Colors indicate samples, shapes indicate gating methods. Again, most approaches were unbiased with respect to central manual gating for the T-cell panel and showed similar variability to central manual gating, with the exception of EMCytoM, which exhibited bias for Activated CD8 T-cells, Central Memory CD4 T-cells, activated CD4 T-cells, and DENSE2, which exhibited bias for the Central Memory Cd8 and Effector memory CD8 T-cells.

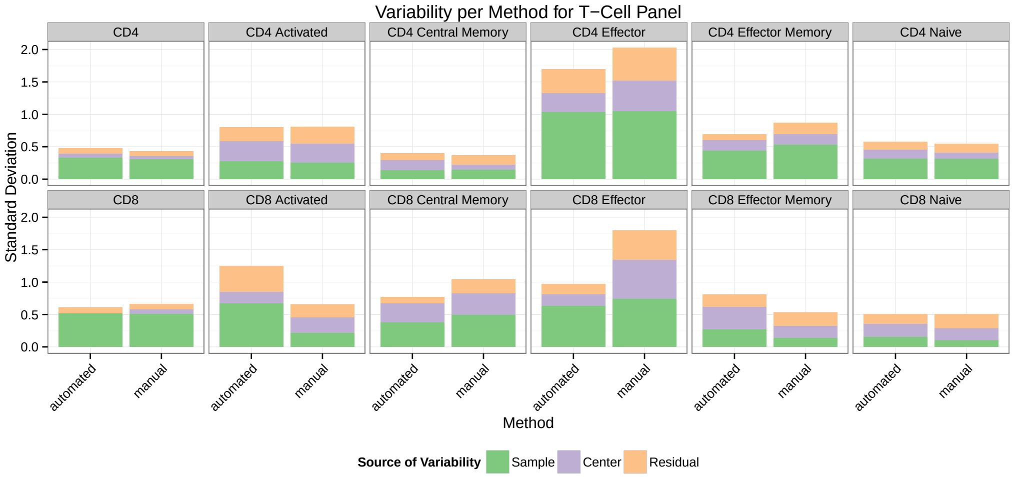


Supplementary Figure 7: Center, biological and residual variability per population and gating method for the T-cell panel. Biological variability dominates across most cell subsets. Automated gating variability is comparable to manual gating variability or decreased across other cell subsets, except the CD8 effector memory and CD8 activated cells. Y-axis is the standard deviation of the center, sample and residual components estimated from the random effects model.

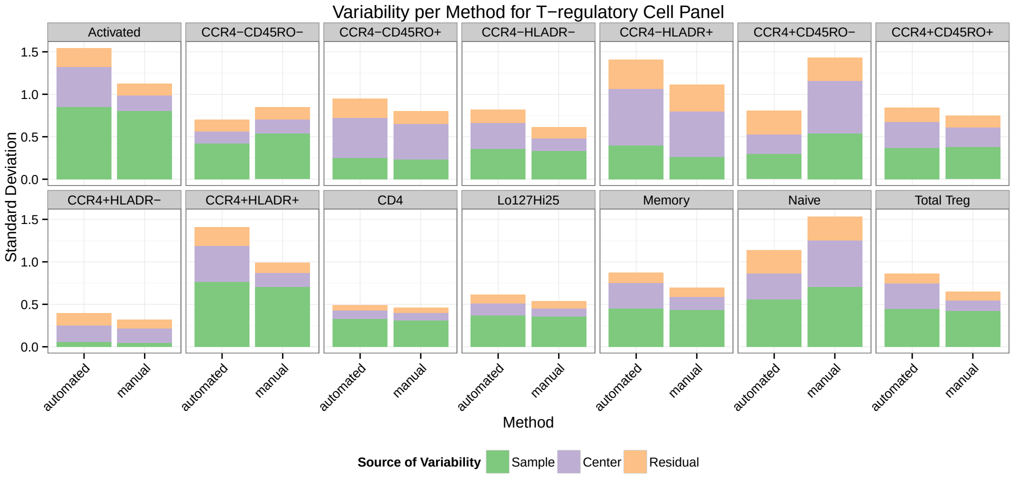


Supplementary Figure 8: Center, biological and residual variability per population and gating method for the T-reg panel. Biological variability dominates technical (center-to-center) variability for most cell subsets. Central manual gating was optimized to minimize the cross-center CV. Y-axis is the standard deviation of the center, sample and residual components estimated from the random effects model.

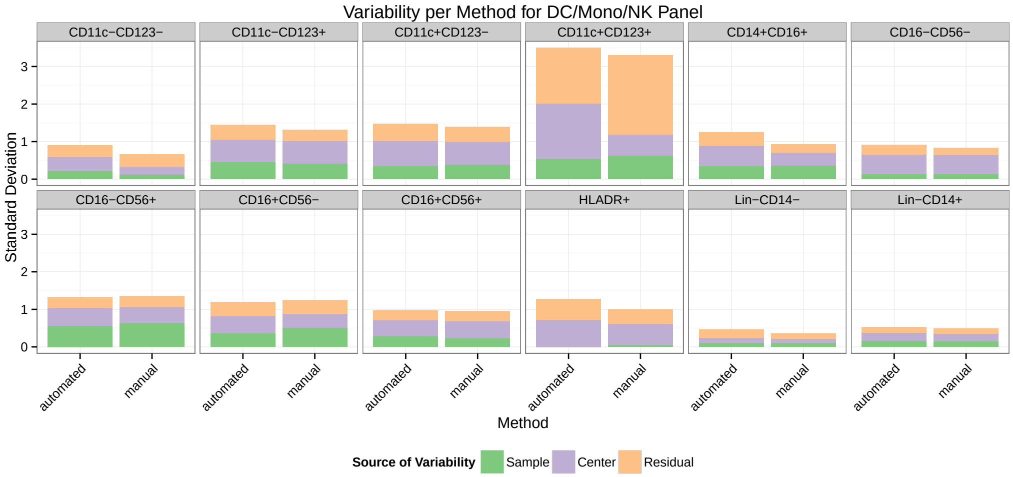


Supplementary Figure 9: Center, biological and residual variability per population and gating method for the DC / Mono / NK panel. Center to center variability dominates across across nearly all cell subsets. The scale of the total variability is comparable between manual and automated gating methods. Central manual gating was optimized to minimize the cross-center CV. Y-axis is the standard deviation of the center, sample and residual components estimated from the random effects model.

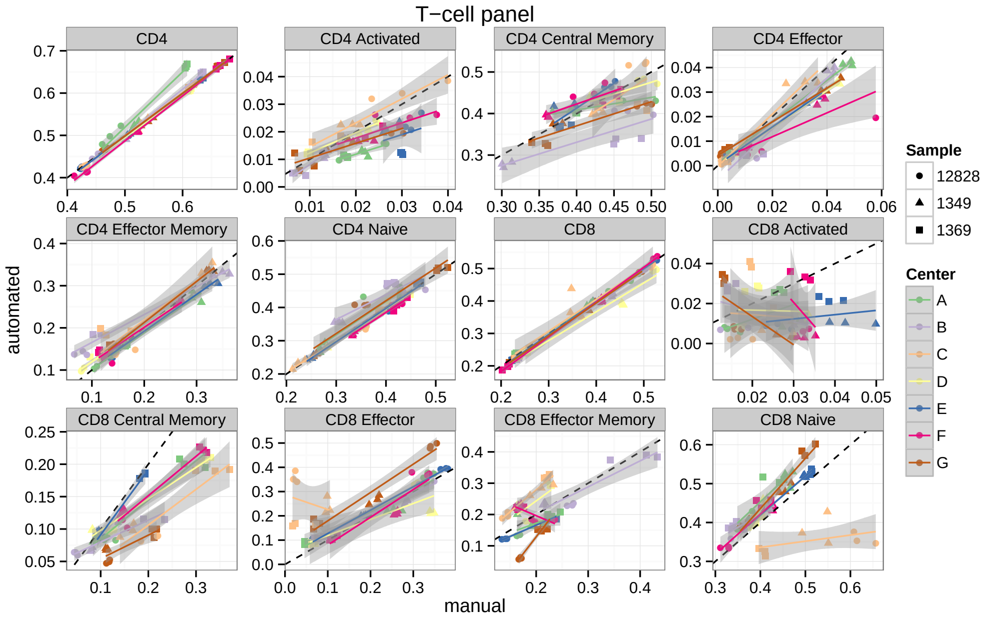


Supplementary Figure 10: Correlation between manual and automated gating for each center and population in the T-cell panel. Poor agreement between automated and manual gating is obvious in the CD8 Activated cell population (a rare cell population), and is reflected in the higher variability for this cell subset. Poor correlation between manual and automated gating in the CD8 Naive cell subset for center C can be traced to sample quality issues, possibly due to compensation problems for the CCR7 and CD45RA markers. Lines show least squares best fit. Shaded region shows the 95% confidence interval of the fit. Dotted line denotes the diagonal $y = x$.

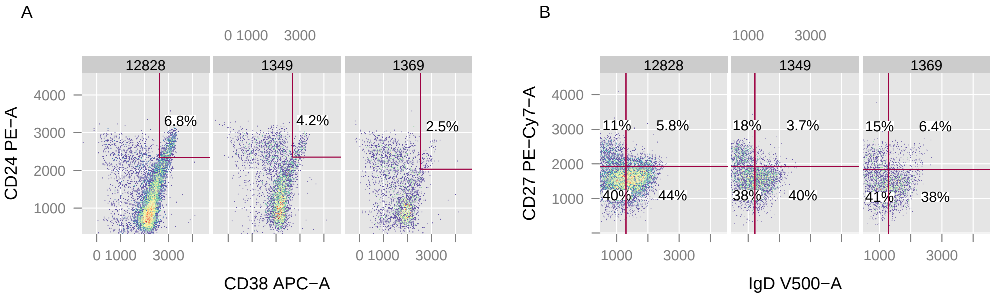


Supplementary Figure 11: An example of central manual gates for cell populations with no clear demarcation of positive and negative cells for population boundaries (transitional cells, CD38high, CD24high) and cell populations that are poorly resolved (IgD+/- CD27+/-) from three different biological samples (rows) from center G. There is little information in the data to discriminate IgD+/- cells in a data-driven fashion due to the poor resolution of the IgD marker, inducing bias and increased variation relative to manual gating. In contrast, manual gates were chosen to minimize the variability across replicates which underestimates the true variability in the data.

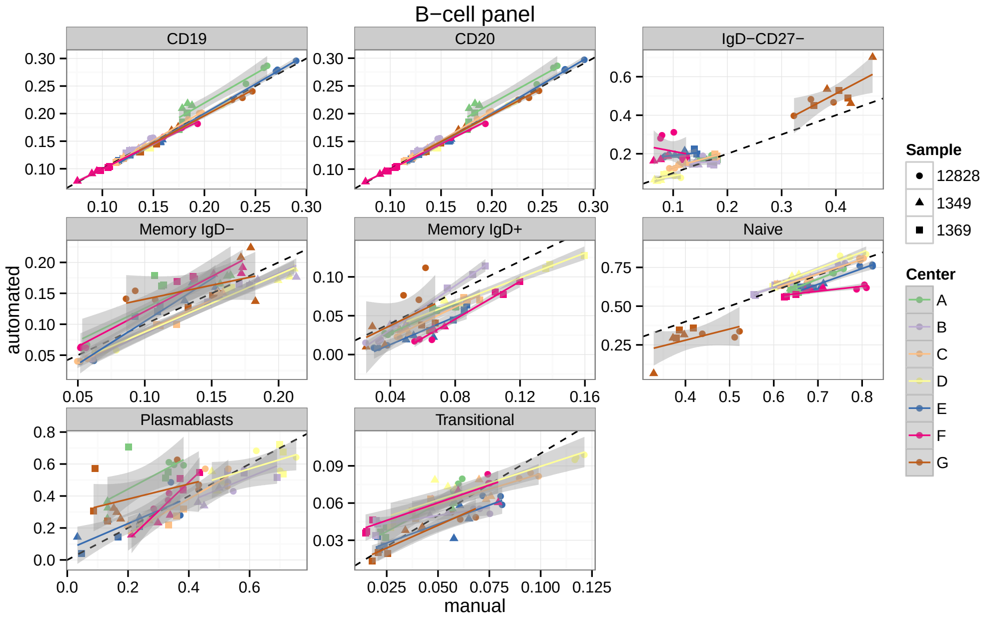


Supplementary Figure 12: Correlation between manual and automated gating for each center and population in the B-cell panel. Lines show least squares best fit. Shaded region shows the 95% confidence interval of the fit.Dotted line denotes the diagonal $y = x$.

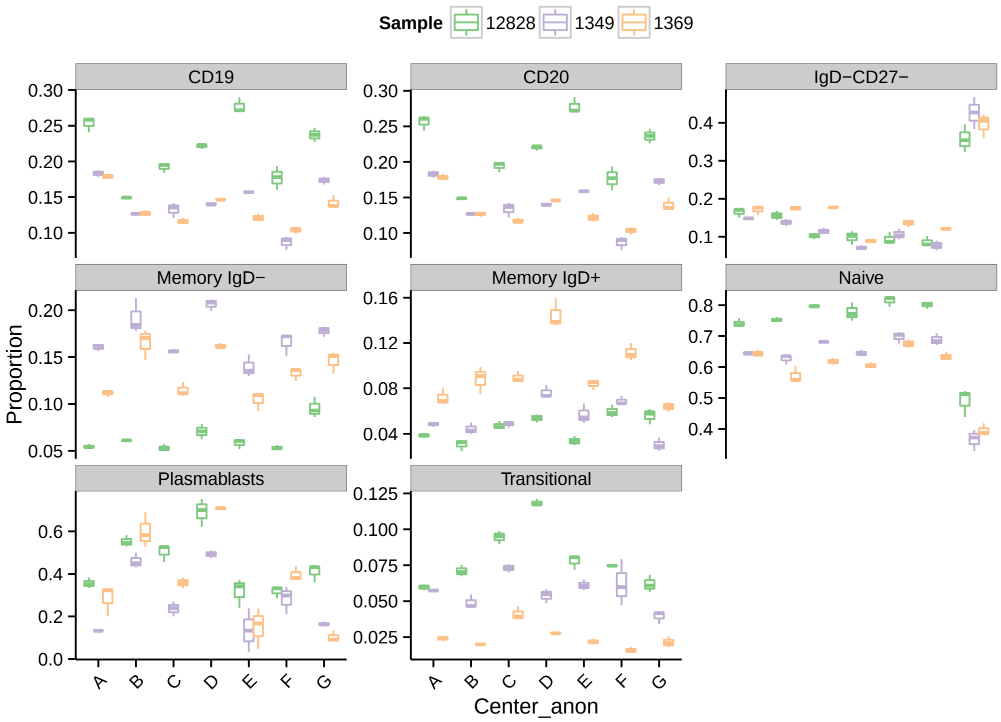


Supplementary Figure 13: Box plots of centrally manually gated cell population proportions for the B-cell panel for each sample and center. Center G is a clear outlier for the Naive, and IgD-CD27- cell populations across the three samples. There is also substantial center to center variability in the plasmablast population (a rare cell subset).

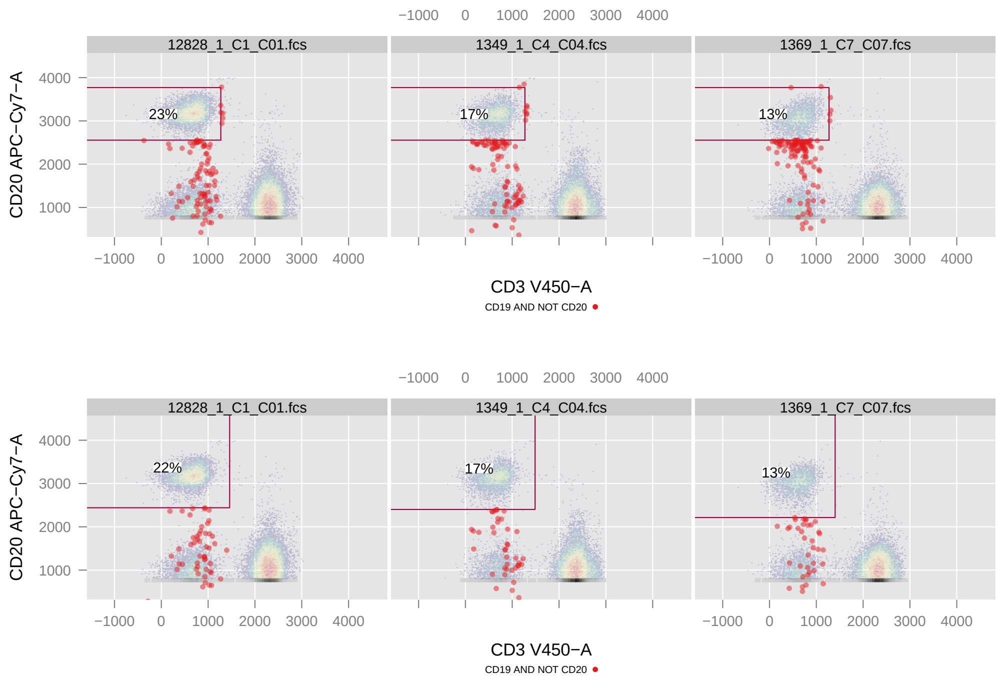


Supplementary Figure 14: Manual and automated gating of CD20+ cells in three biological samples from the B-cell panel from center G. B-cells are gated as CD19 and not CD20 and CD3-. The overlay shows the CD19 and not CD20 population. The manual gates (top row) do not adequately capture all the CD20+ cells, while the OpenCyto automated gates (bottom row) exclude the CD20+ cells from the B-cell population. This impacts the denominator of the plasmablast population proportion estimate, introducing a bias in the population statistics, even though the plasmablast manual and automated gates are comparable. The OpenCyto CD20 gate is more accurate, resulting in lower within-subject variability in the Plasmablast population in Figure 4.

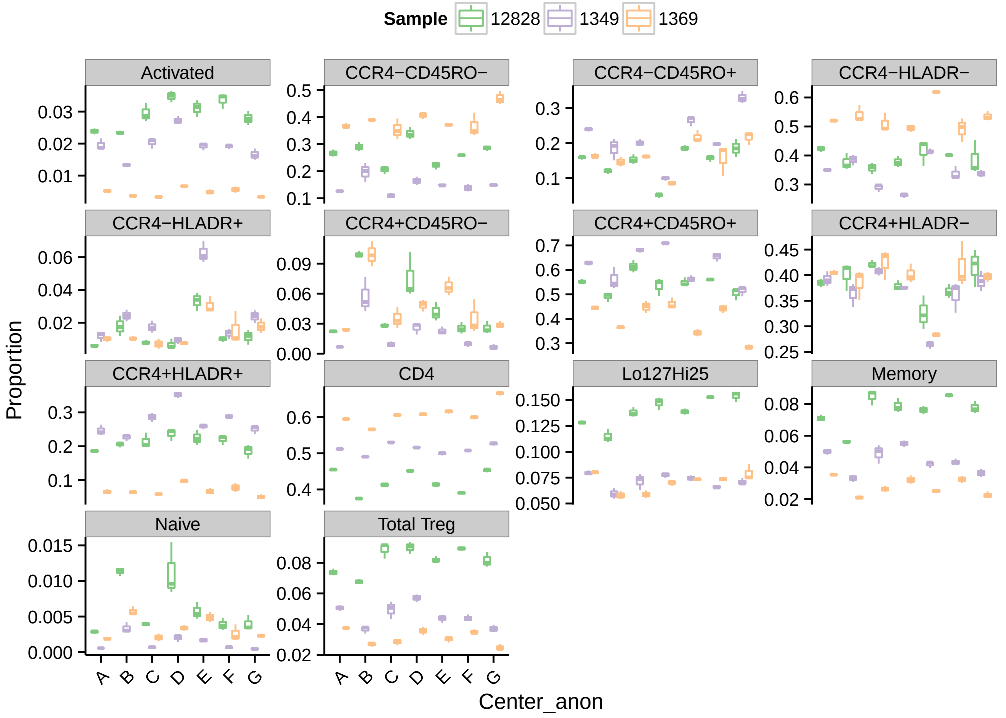


Supplementary Figure 15: Boxplots of centrally manually gated cell population proportions for the T-regulatory panel for each sample and center. Some outliers can be observed, such as the Naive cell population in sample 12828 from center D. There is also large center-to-center variability, relative to biological variation, in CCR4-HLADR+, CCR4+CD45RO-, CCR4+HLADR- cells.

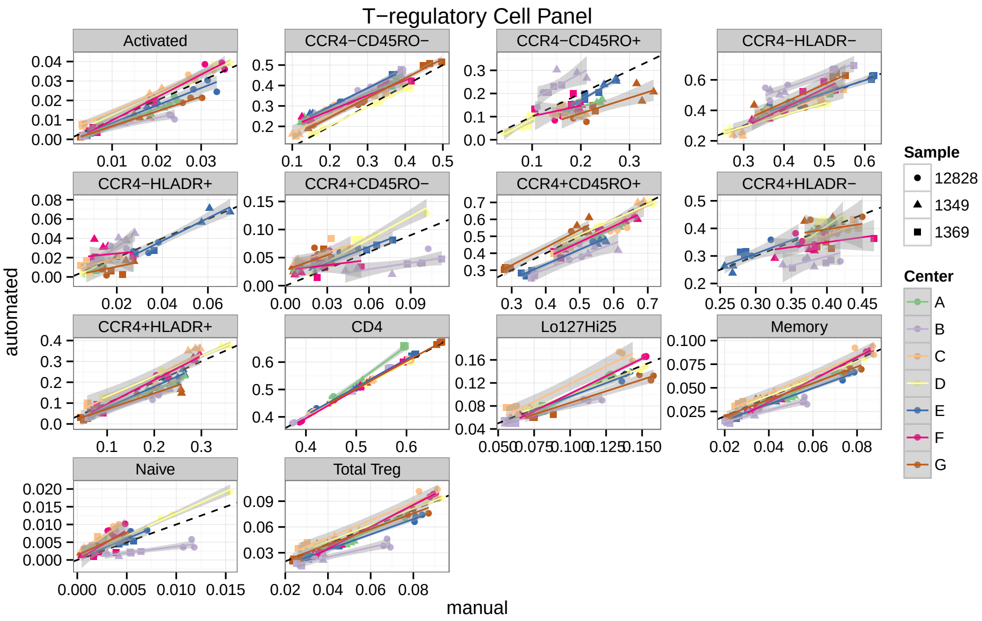


Supplementary Figure 16: Correlation between manual and automated gating for each center and population in the T-regulatory cell panel. Discrepancies in the CCR4+HLADR- cell population are due in part to poor resolution in the CCR4 marker, but also because the parent population is relatively low abundance (mean = 2288 cells, sd = 1242, range = [605, 6089], across all samples and centers). Center B exhibits discrepancies across multiple cell populations, which are boolean combinations of the problematic CCR4 containing subsets. Lines show least squares best fit. Shaded region shows the 95% confidence interval of the fit. Dotted line denotes the diagonal $y = x$.

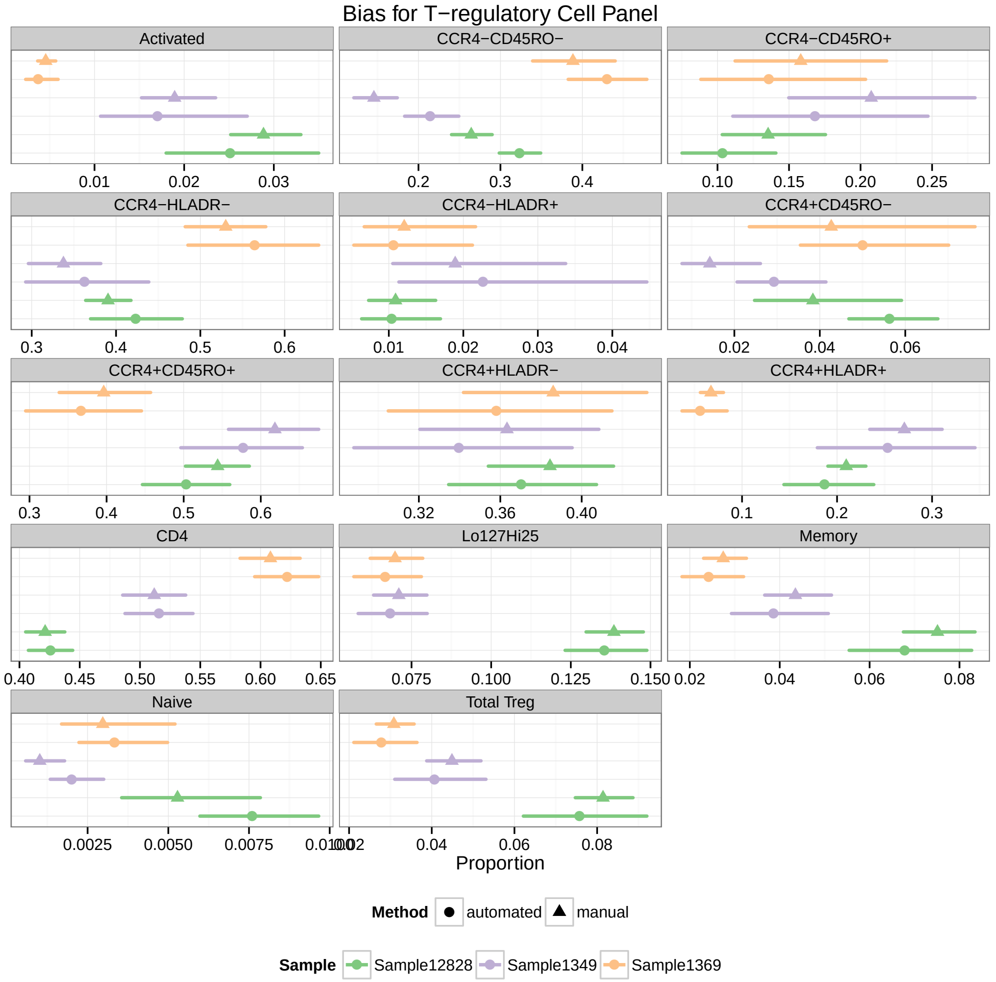


Supplementary Figure 17: Estimated cell proportions from each population and gating method in the T-regulatory panel. Estimated proportions and 95% confidence intervals are shown for each sample, gating method, and cell population in the T-regulatory panel. There are no significant differences between the estimates from the automated gating methods compared to central manual gating.

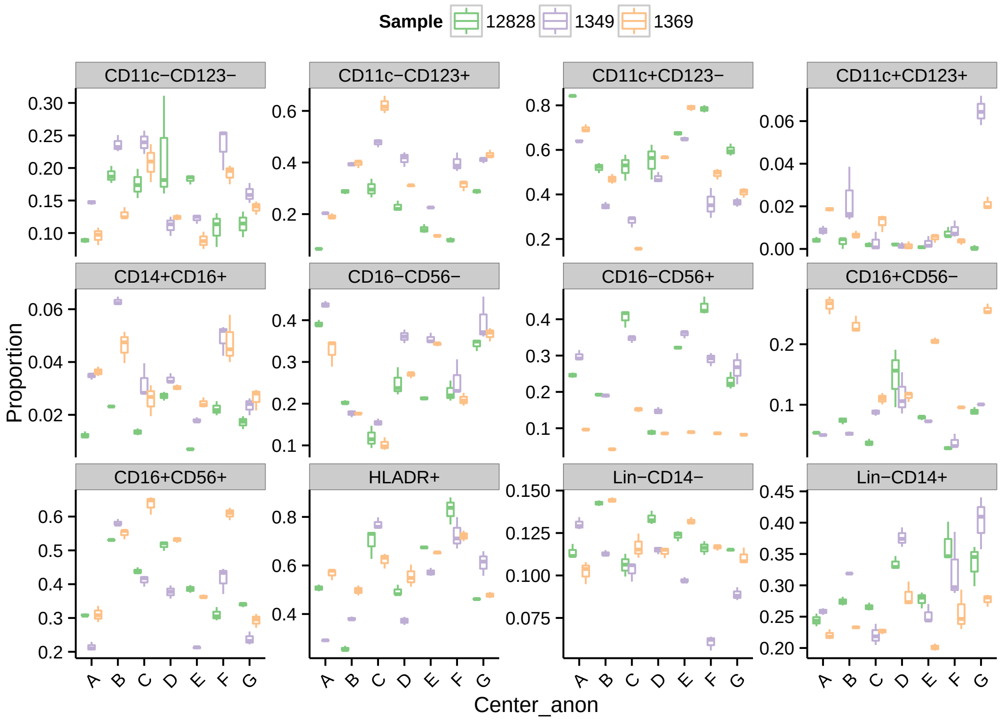


Supplementary Figure 18: Boxplots of centrally manually gated cell population proportions for the DC / Monocyte / NK panel for each sample and center. There is large center to center variability that masks the biological variability between samples for all cell populations.

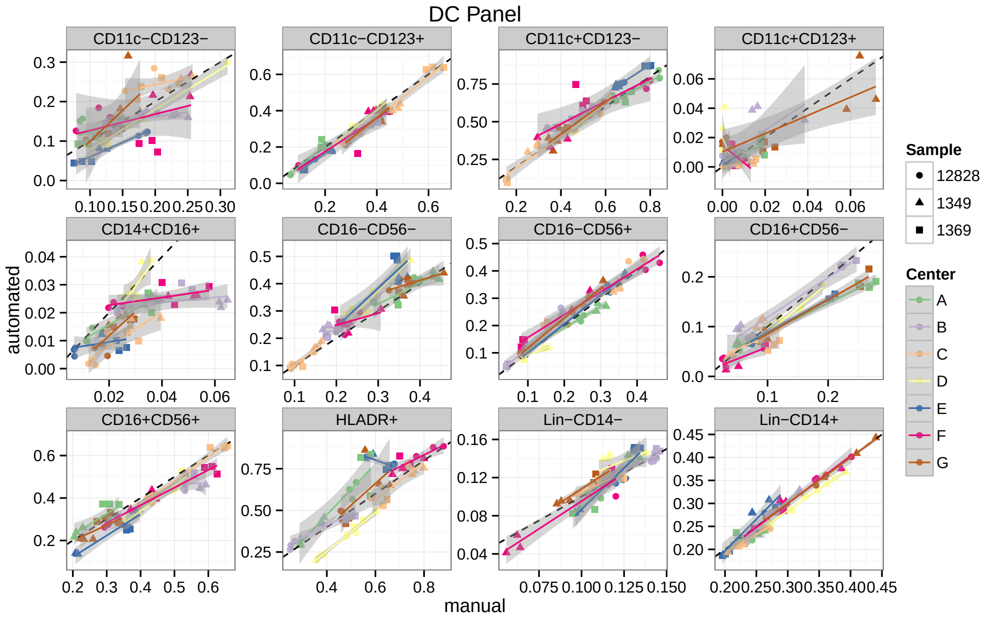


Supplementary Figure 19: Correlation between manual and automated gating for each center and population in the DC panel. There is relatively good agreement between manual and automated gating for all cell populations, with some increased variablility for the CD11c-CD123- population. Lines show least squares best fit. Shaded region shows the 95% confidence interval of the fit. Dotted line denotes the diagonal $y = x$.

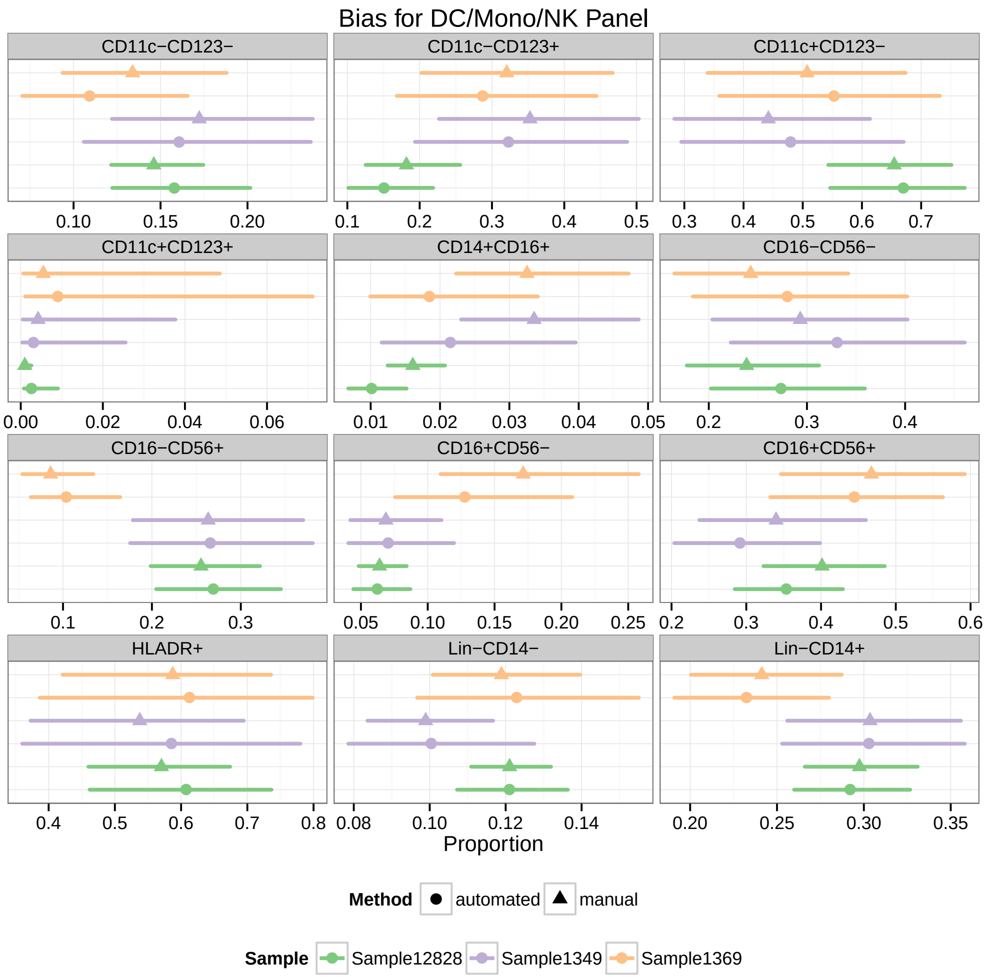


Supplementary Figure 20: Estimated cell proportions from each population and gating method in the DC / Mono / NK panel. Estimated proportions and 95% confidence intervals are shown for each sample, gating method, and cell population in the DC / Mono / NK panel. Large technical (center-to-center) variation is evident from the wide confidence bands for all methods compared to the biological variation across the majority of populations.

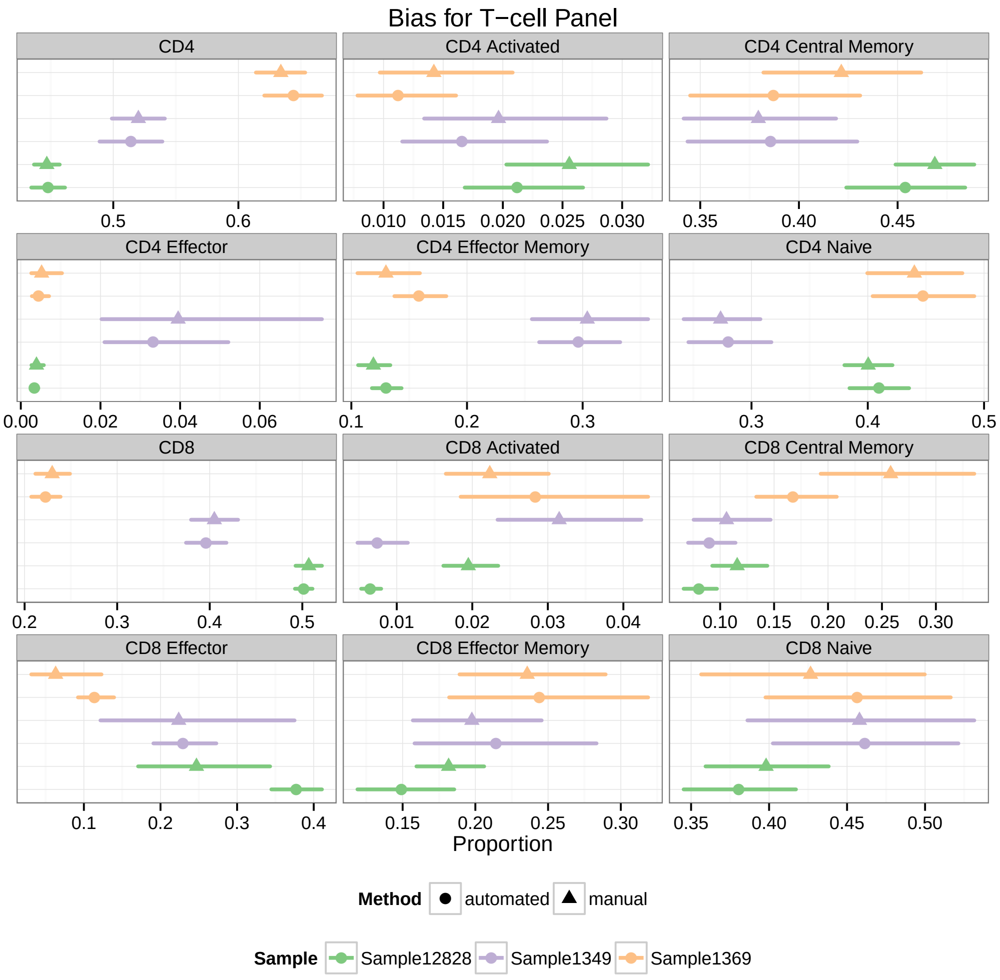


Supplementary Figure 21: Estimated cell proportions from each population and gating method in the T-cell panel. Estimated proportions and 95% confidence intervals are shown for each sample, gating method, and cell population in the T-cell panel. Sample 12828 shows considerable variation between different gating methods for several CD8 cell subsets. The flowDensity and OpenCyto automated methods exhibit narrower confidence bands than manual gating for the CD8 effector and CD4 central memory cell subsets. When flowDensity and OpenCyto have biased estimates, the bias is small relative to the biological variation and the methods tend to agree between each other. The CD8 effector memory subset is problematic for all methods.

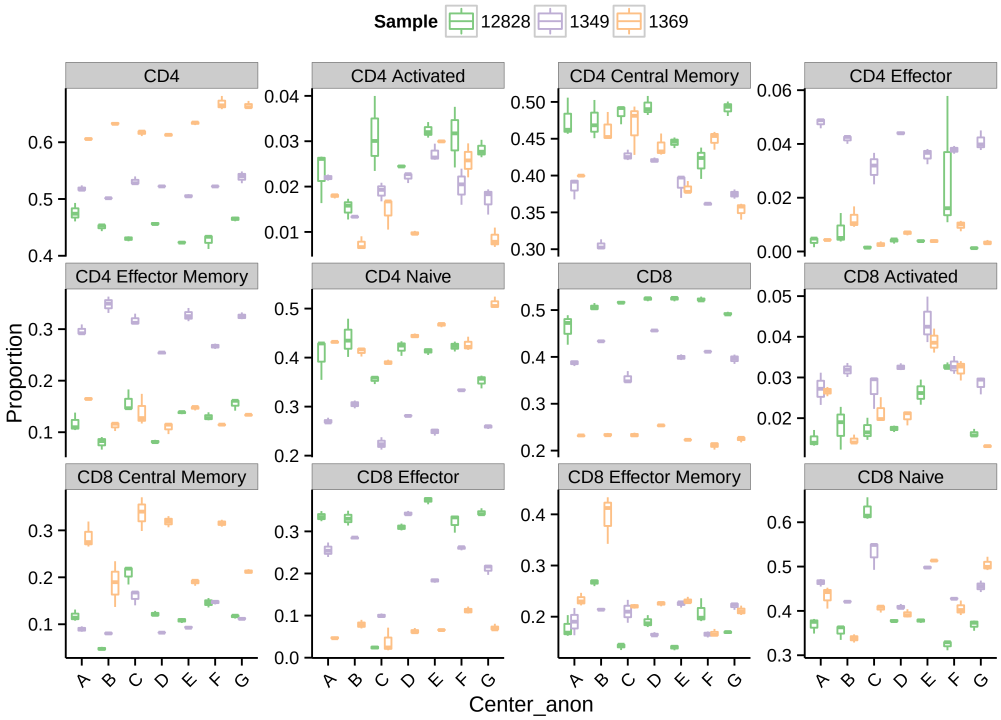


Supplementary Figure 22: Boxplots of centrally manually gated cell population proportions for the T-cell panel for each sample and center. Several outliers can be observed across different centers and cell populations. Most notably, the CD4 effector population in sample 12828 from center F has large variability relative to the other samples and centers. Similarly, a bias is observed in sample 1369 from center B in the CD8 Effector Memory population. CD4 activated cells show large variation within samples across most centers.

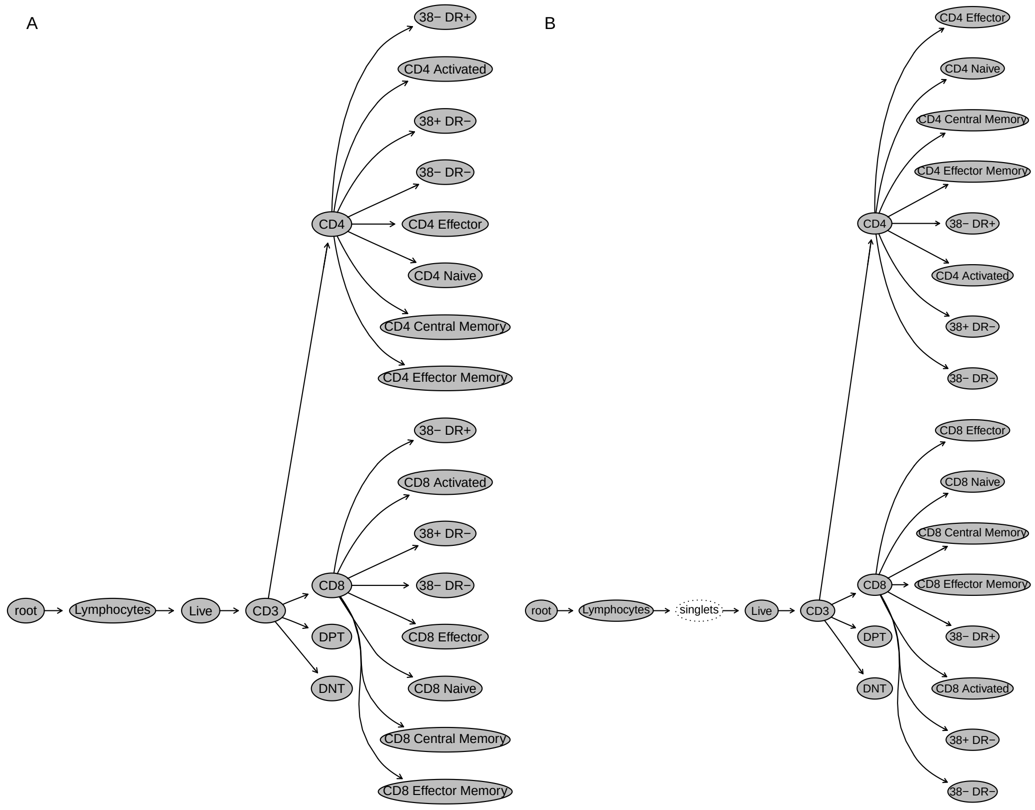


Supplementary Figure 23: Central manual gating schemes for the T-cell panel for Center B (left) and a representative compliant center (right). Center B did not collect channel information to allow singlet gating.

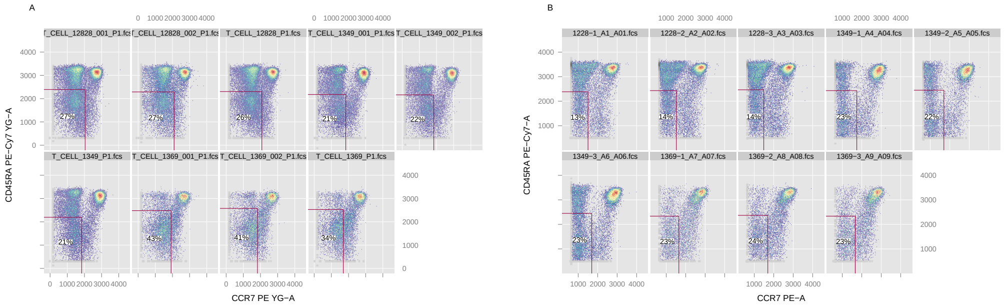


Supplementary Figure 24: Central manual gates of CD8 Effector memory cells for center B (top), and a representative compliant center (bottom). The differences in the distribution of the CCR7 marker is clear, with poor resolution of positive and negative cells in CCR7 for Center B, compared to the compliant center.

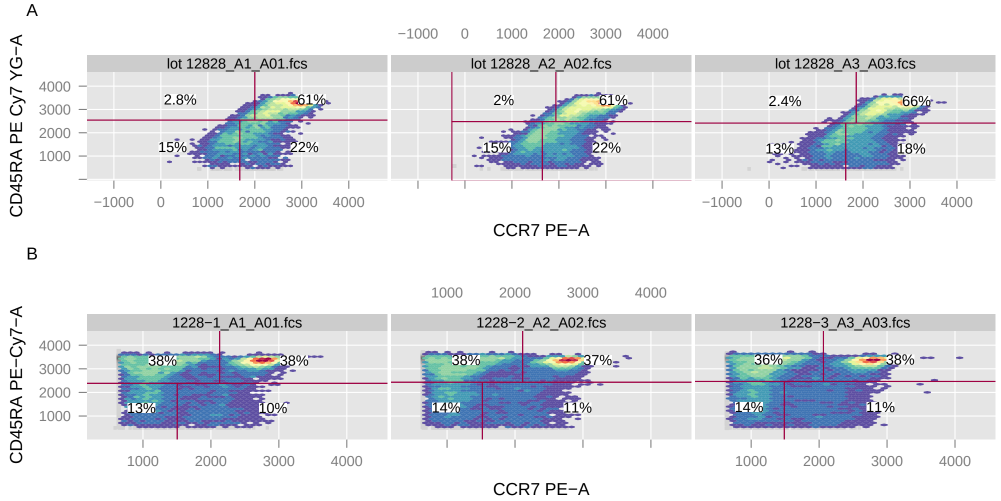


Supplementary Figure 25: Example of center variability in the CD8 T-cell panel for center C (top) with compensation problems on CD197 (CCR7) and CD45RA leading to bias in the automated analysis. A representative sample from a non-problematic center is shown (bottom). Discrimination of the CD45RA population is difficult in the samples from Center C, possibly due to problems with compensation.

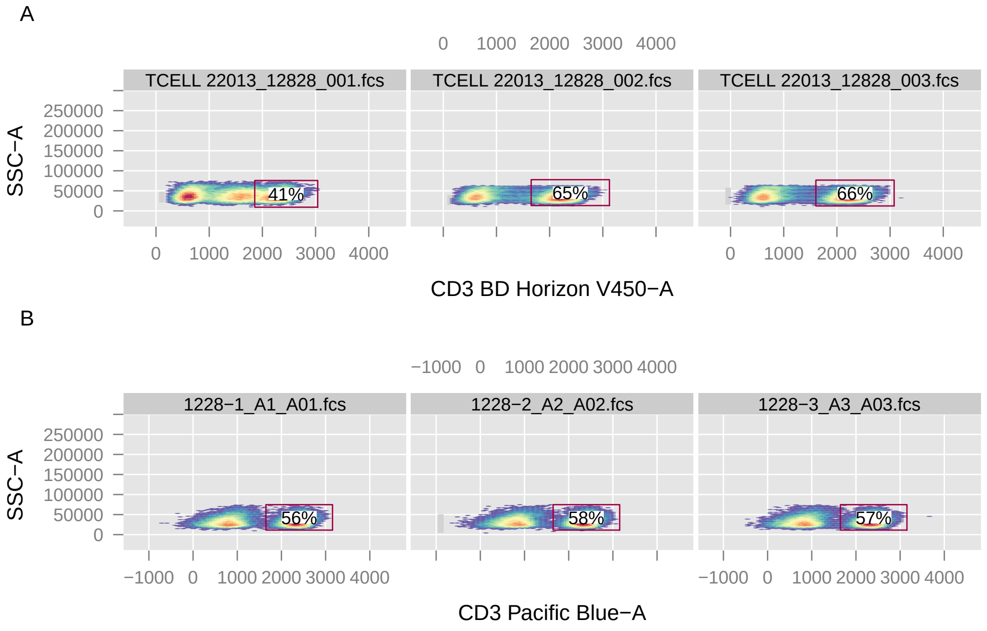


Supplementary Figure 26: Variation in CD3 distribution of center F sample 12828 compared to a representative center (bottom). The distribution of CD3 exhibits a trimodal distribution for one of the sample 12828 replicates. Although the cause is unclear, such oddities may account for the variability in the CD4 activated population estimates for this sample.

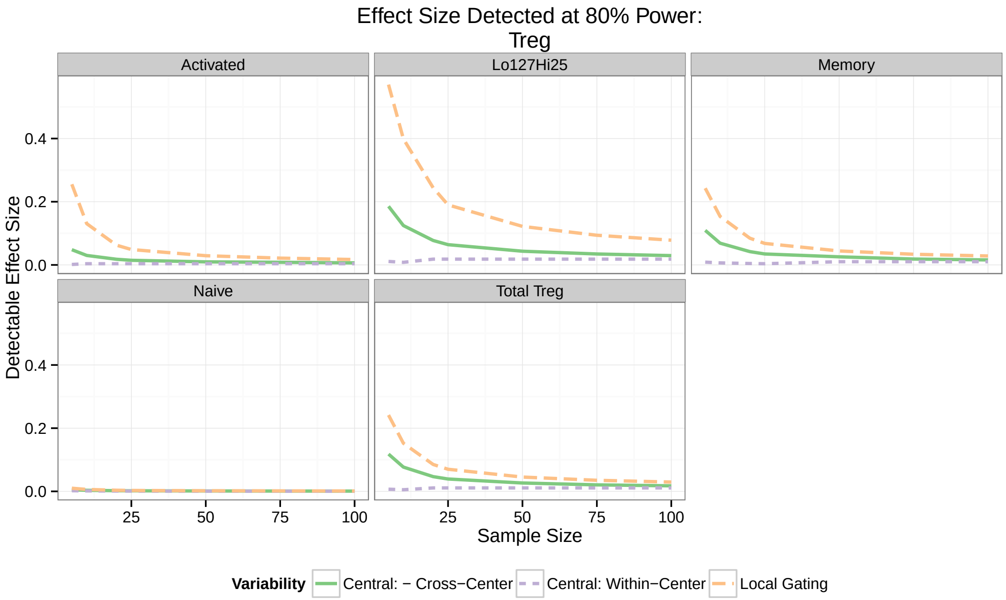


Supplementary Figure 27: Power analysis for the T-regulatory cell panel. The main increase in power comes from centralized gating.

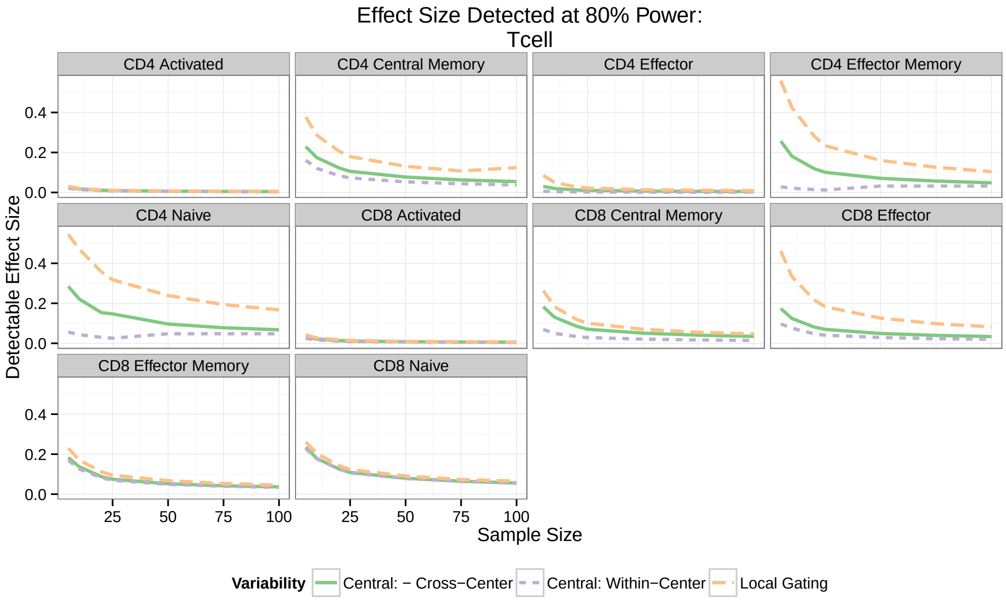


Supplementary Figure 28: Power analysis for the T-cell panel. the main benefit comes from centralized gating, with a few cell subsets exhibiting significant center-to-center variability (CD4 Effector, CD4 Central Memory, CD8 Central Memory).

# 2 Power analysis

Here, we provide an alternative interpretation of the power analysis presented in the paper. The power analysis can be considered as follows. If one envisions a hypothetical experiment where one is measuring the proportion of cells in a cell population, and that proportion changes in the hypothetical treatment group vs. the control group, then the size of that change is termed the ?effect size?. The power analysis curves show the smallest effect size that can be reliably detected (by reliably, we mean detected with 80% probability if it is present), as a function of sample size (i.e. the size of each treatment and control group), and as a function of the type of variation present in the data (i.e. 1. if the experiment was performed at a single center with local gating, 2. If the experiment was performed at multiple centers with central gating, and 3. If the experiment was performed at multiple centers with local gating at each center). A larger minimum detectable effect size is indicative of more variable estimates of the population effects in the treatment and control groups. The estimates of variability due to central vs. local gating and due to differences center-to-center were estimated from the data study data. The power analysis couches the study results in terms of sample size vs. effect size estimates that can be applied in practice for the design of new experiments.

# 3 ImmuneSpace

All code and data are hosted on http://www.immunespace.org and are accessible under the username: “HIPCLyoplate@immunespace.org” password: “reproducibility”

# 4 Lyoplate Protocol

# Phenotyping of Human PBMC using Lyoplate

## 1. Principle

In this protocol, we use lyophilized cocktails of fluorescent-labeled antibodies preconfigured in 96-well plates (Lyoplates) to stain PBMC that have been previously cryopreserved. We use a BD digital flow cytometer to acquire the fluorescence data. Subsequent analysis of the fluorescence data using FlowJo software allows for cell types to be analysed based on cell size, internal complexity, and relative fluorescence intensity in each color. The percentage of each cell type is determined and reported as a percent of the parent cell type.

## 2. Materials and Equipment

2.1. PBMC, fresh or thawed frozen
2.2. Complete RPMI (RPMI with 10% FBS, P/S, glutamine)
2.3. 2% Para-formaldehyde (PFA) in PBS, made fresh before use
2.4. Phosphate buffered saline (PBS)
2.5. FACS buffer (PBS with 2% FBS and 0.1% Na Azide)
2.6. Phenotyping antibodies
2.7. 37°C water bath
2.8. 96-well plate
2.9. Biosafety cabinet
2.10. Centrifuge
2.11. Calibrated pipettes
2.12. ViCell or Hemocytometer cell counter
2.13. Invitrogen Live/Dead Green dye (L23101)

## 3. Procedure

### Viability Dye Preparation

3.1 Bring one vial of the fluorescent reactive dye (Component A) and the vial of anhydrous DMSO (Component B) to room temperature before removing the caps.
3.2 Add 50 µL of DMSO to the vial of reactive dye. Mix well and

visually confirm that all of the dye has dissolved.

3.3 Use the solution of reactive dye as soon as possible, ideally within a few hours of reconstitution (once reconstituted, the DMSO solution of reactive dye is somewhat unstable, especially if exposed to moisture. Unused portions may be used for up to 2 weeks if stored at ≤–20°C, protected from light and moisture).

**PBMC thawing**

1. Use a dry ice tub to transfer vials to the lab, making sure to keep the vials submersed in the dry ice to keep cold.
2. Hold the frozen vial in water in a 37 ± 3°C water bath.
   a. GENTLY shake the vial in the water until just before the last ice crystal has melted.
   b. Check to make sure the cap is secure. It is possible that the cap may loosen slightly with the temperature change between the ice and the water bath.
   c. Remove the vial from the water after the last ice crystal has been melted for approximately 1 minute.
3. Wipe the vial with an alcohol pad, focusing on the cap area.
4. Slowly unscrew the cap as pressure may build inside tube.
5. Add 1 mL of culture media that has been brought to 37 ± 3°C (warm) to the vial using a 1000 µL pipette tip and drop-wise action. Add the culture media slowly to allow the cells to adjust to the change of temperature.
6. Slowly add the cells to a 50 mL conical tube containing 8 mL of warm culture media, using the same 1000 µL pipette tip. The gradual dilution of DMSO, contained in the freezing media, avoids osmotic shock and the warm temperature assures that the cells can actively compensate for the osmotic pressure.
7. Add 1 mL of warm culture media to the original vial to recover cells that may have adhered to the sides; add to the 50 mL conical tube.
8. Pellet the cells by centrifugation at 1200-1400 rpm for 10 minutes with rapid acceleration and brake on.
9. Discard supernatant. Re-suspend the cell pellet by gently tapping (avoid excessive shear forces) while adding 5 mL of warm culture media.

10. Count cells using laboratory specific procedures and dilute to 10^6 cells/mL, if there are more than 5x10^6 total cells.

**Viability dye staining**

3.4 Dilute the reconstituted live/dead dye (from step 3.2) 1:50 by adding 10 ul to 490 ul of PBS. Then add 2 ul of this diluted solution per ml of cell suspension. Invert to mix.

3.5 Incubate at room temperature for 30 minutes, protected from light.

3.6 Add 10 ml of FACS buffer, centrifuge at 350 x G for 8 minutes at room temperature.

3.7 Remove supernatant from the cells and resuspend the pellet by tapping the tube. Add FACS buffer to a cell concentration of $10^7$ cells/ml (a minimum of 250 µl are needed to stain all wells of the Lyoplate, for a single replicate; 750 µl are needed for all 3 replicates).

**Cell staining**

3.8 Add 50-60 µl of cells from each sample into the first 5 wells (A-E) of each sample column on the Lyoplate. Add cells to the side of each well without touching pellets and let sit for 2-3 mins, then mix up and down 5-6 times using a multichannel pipette. Avoid creating bubbles. Add the cells for lot 12828 to columns 1-3; add the cells for lot 1349 to columns 4-6; and add the cells for lot 1369 to columns 7-9. You can use columns 10-12 for your own controls or leave them blank.

3.9 Add 50 µl of FACS buffer to the compensation control wells (F1 through H5).

3.10 Incubate 45 minutes at room temperature with gentle shaking.

3.11 Follow these steps for all wells used, including compensation controls: Add 150 µl of FACS buffer, without pipetting up and down, and spin at 1200 rpm, 2min, @ 4˚C, for the first wash. Aspirate, resuspend in 200 µl FACS buffer & spin 1200 rpm, 2 min, @ 4˚C, for a second wash. Repeat washing with 200 µl FACS buffer for a third and fourth wash. After the 4th wash, resuspend wells in 200 µL FACS buffer, cover with foil and keep at 4˚C until ready to run on LSR II. Alternately, after two washes, resuspend in 200 uL of 2% PFA in PBS, incubate 15 minutes, then complete the last two washes as above.
Create a new blank experiment using baseline CST settings, then run single-color controls and adjust voltages to achieve specified

target channels. Adjust the FSC voltage gain so that the lymphocyte population is centered around 100,000; ensure that the FSC threshold has eliminated most of the debris but does not cut into the lymphocytes.

| COMP CONTROL | Min MFI | TARGET MFI | Max MFI |
|---|---|---|---|
| GREEN LIVE | 18,000 | 20,000 | 22,000 |
| CD197 PE | 54,000 | 60,000 | 66,000 |
| CD4 PerCP Cy5.5 | 2,250 | 2,500 | 2,750 |
| CD194 PE Cy7 | 27,000 | 30,000 | 33,000 |
| CD38 APC | 22,500 | 25,000 | 27,500 |
| CD20 APC-H7 | 22,500 | 25,000 | 27,500 |
| CD3 V450 | 6,750 | 7,500 | 8,250 |
| HLA DR V500 | 13,500 | 15,000 | 16,500 |

3.12 If using HTS, settings should be: Throughput mode: standard; Sample flow rate 1.0; Sample volume (ul): 200; Mixing volume (ul): 100; Mixing speed (ul/sec): 180; Number of mixes: 3; Wash volume (ul): 400.

3.13 Labeling and collection criteria:

- Turn on Area, Height, and Width parameters for FSC and SSC; and Area for all other labeled channels.
- Set the FSC voltage gain so that lymphocytes are near the center of the scale, and keep the FSC threshold low enough that some of the debris is visible.
- Name all markers as they are labeled in the figure on p 6. Don't abbreviate (e.g., use "CD19 PerCP-Cy5.5", not "19 PCP", etc.).
- Fill out the excel template provided to allow mapping of experimental and comp control samples to files.

3.14 Name the specimens by donor ID and replicate, e.g., 1349_1.fcs, 1349_2.fcs, etc. Record the compensation specimen, then calculate compensation, and record the experimental samples. Set as stopping criteria: 50,000 live CD3+ cells for the T cell, Treg, and Th1/2/17 tubes; 20,000 live CD20+ cells for the B cell tubes; and 20,000 live CD14+ cells for the DC/mono/NK tubes.

**Data analysis**

3.15 Manually check and adjust compensation if needed, by viewing a grid of dot plots where all color combinations are plotted for a sample gated only on lymphocytes (use bioexponential scaling to help find compensation problems if there are any).

3.16 Analyze data with FlowJo for Mac software, if available, creating gates for the following cell subsets:

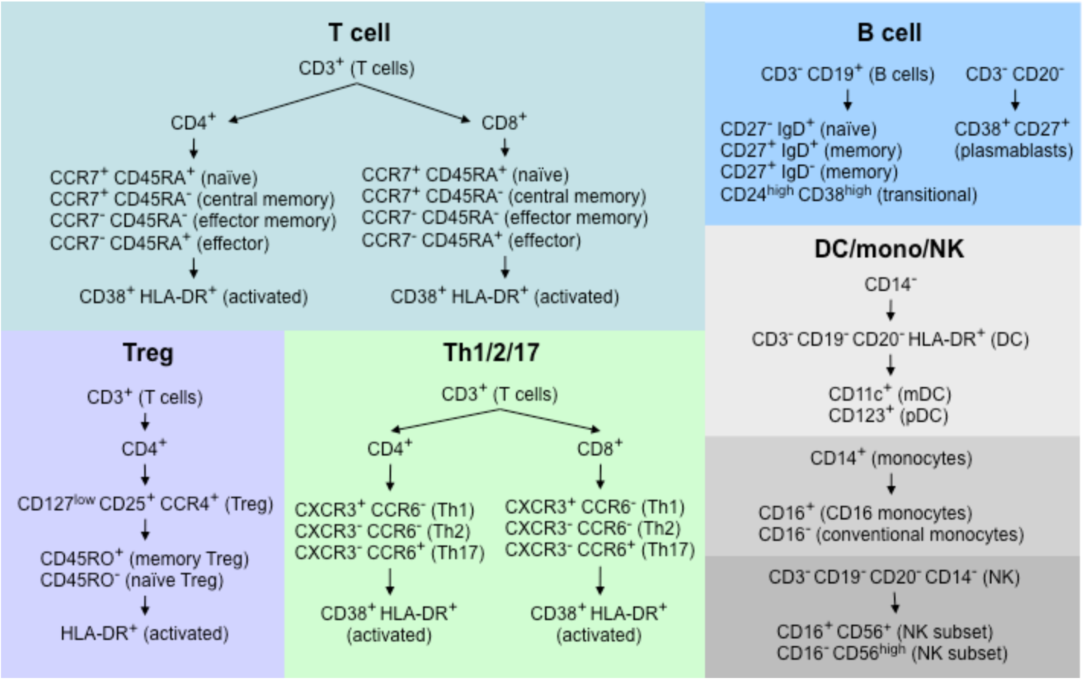


3.17 Adjust gates on a donor-specific basis, if necessary, to control for any differences in background or positive staining intensity.

3.18 Export % parent statistics for each gated population to an Excel spreadsheet. Post the spreadsheet, mapping file, FlowJo workspace, and fcs files to Basecamp as a single zipped file.

**BD Lyoplate™**

Mat No: 623882
Lot No: 35286
Exp: 2013-08-31

**Stain 80 CTT***

94 Tests
Store at 16-29°C

| | 1 | 2 | 3 | 4 | 5 | 6 | 7 | 8 | 9 | 10 | 11 | 12 | |
|---|---|---|---|---|---|---|---|---|---|---|---|---|---|
| A | CD197 PE / CD4 PerCP-Cy™5.5 / CD45RA PE-Cy™7 / CD38 APC / CD8 APC-H7 / CD3 V450 / HLA-DR V500 | | | | | | | | | | | | T CELL |
| B | CD25 PE / CD4 PerCP-Cy™5.5 / CD194 PE-Cy™7 / CD127 Alexa®Fluor647 / CD45RO APC-H7 / CD3 V450 / HLA-DR V500 | | | | | | | | | | | | TREG |
| C | CD24 PE / CD19 PerCP-Cy™5.5 / CD27 PE-Cy™7 / CD38 APC / CD20 APC-H7 / CD3 V450 / IgD V500 | | | | | | | | | | | | B CELL |
| D | CD56 PE / CD123 PerCP-Cy™5.5 / CD11c PE-Cy™7 / CD16 APC / CD3+CD19+CD20 APC-H7 / CD14 V450 / HLA-DR V500 | | | | | | | | | | | | DC/MONO/ NK |
| E | CD183 PE / CD4 PerCP-Cy™5.5 / CD196 PE-Cy™7 / CD38 APC / CD8 APC-H7 / CD3 V450 / HLA-DR V500 | | | | | | | | | | | | TH1/2/17 |
| F | Neg. Beads Only | LIVE GREEN | CD197 PE | CD4 PerCP-Cy™5.5 | CD45RA PE-Cy™7 | CD194 PE-Cy™7 | CD27 PE-Cy™7 | CD11c PE-Cy™7 | CD196 PE-Cy™7 | CD38 APC | CD127 Alexa 647 | CD8 APC-H7 | COMP BEADS |
| G | CD45RO APC-H7 | CD20 APC H7 | CD3+ 19+20 APC-H7 | CD3 V450 | HLA-DR V500 | | | Neg. Beads Only | LIVE GREEN | CD197 PE | CD4 PerCP-Cy™5.5 | CD45RA PE-Cy™7 | EMPTY WELLS |
| H | CD194 PE-Cy™7 | CD27 PE-Cy™7 | CD11c PE-Cy™7 | CD196 PE-Cy™7 | CD38 APC | CD127 Alexa 647 | CD8 APC-H7 | CD45RO APC-H7 | CD20 APC H7 | CD3+ 19+20 APC-H7 | CD3 V450 | HLA-DR V500 | |

| Cocktail: | T CELL | TREG | B CELL | DC/MONO/NK | TH1/2/17 |
|---|---|---|---|---|---|
| | | | | | |
| FITC | open | open | open | open | open |
| PE | **CCR7**<br>BD 560765<br>(150503) | **CD25**<br>BD 341009<br>(2A3) | **CD24**<br>BD 555428<br>(ML5) | **CD56**<br>BD 555516<br>(B159) | **CXCR3**<br>BD 557185<br>(1C6/CXCR3) |
| PERCP-CY5.5 | **CD4**<br>BD 341654<br>(SK3) | **CD4**<br>BD 341654<br>(SK3) | **CD19**<br>BD 340951<br>(SJ25C1) | **CD123**<br>BD 558714<br>(7G3) | **CD4**<br>BD 341654<br>(SK3) |
| PE-CY7 | **CD45RA**<br>BD 337167<br>(L48) | ***CCR4****<br>*BD 557864*<br>*(1G1)* | **CD27**<br>BD 560609<br>(M-T271) | **CD11c**<br>BD 561356<br>(B-LY6) | **CCR6**<br>BD 560620<br>(11A9) |
| APC | **CD38**<br>(HIT2) | **CD127**<br>BD 560905<br>(HIL-7R-M21) | **CD38**<br>(HIT2) | **CD16**<br>BD 561304<br>(B73.1) | **CD38**<br>(HIT2) |
| APC-H7 | **CD8**<br>BD 560179<br>(SK1) | **CD45RO**<br>BD 561137<br>(UCHL1) | **CD20**<br>BD 560734<br>(2H7) | **CD3+CD19+CD20**<br>BD 560176 (SK7)<br>BD 643078 (SJ25C1)<br>BD 560734 (2H7) | **CD8**<br>BD 560179<br>(SK1) |
| V450 | **CD3**<br>BD 560365<br>(UCHT1) | **CD3**<br>BD 560365<br>(UCHT1) | **CD3**<br>BD 560365<br>(UCHT1) | **CD14**<br>BD 560349<br>(MPHIP9) | **CD3**<br>BD 560365<br>(UCHT1) |
| V500 | **HLA-DR**<br>BD 561224<br>(G46-6) | **HLA-DR**<br>BD 561224<br>(G46-6) | **IgD**<br>BD 561490<br>(IA6-2) | **HLA-DR**<br>BD 561224<br>(G46-6) | **HLA-DR**<br>BD 561224<br>(G46-6) |

# 5 Cytotrol Protocol

# Analysis of Cyto-Trol cells using Lyoplate

## 1. Principle

In this protocol, we use lyophilized cocktails of fluorescent-labeled antibodies preconfigured in 96-well plates (Lyoplates) to stain PBMC that are also lyophilized (Cyto-Trol).

## 2. Materials and Equipment

2.1. Cyto-Trol control cells, 2 vials
2.2. HIPC Lyoplate, 1 plate
2.3. FACS buffer (PBS with 2% FBS and 0.1% Na Azide)
2.4. Centrifuge
2.5. Calibrated pipettes

## 3. Procedure

### Reconstitution of Cyto-Trol Cells

3.1 Resuspend each vial of Cyto-Trol cells with 1 mL of the supplied Reconstitution Buffer. Genly mix by finger-tapping, then let sit for 10 min at room temperature.
3.2 Gently pipet up and down in each vial, then combine the contents of the two vials in a single tube.

### Cell staining

3.3 Carefully peal back the foil cover of the Lyoplate. Add 80 $\mu$l of resusupended Cyto-Trol cells into wells 1A-4E in the Lyoplate. This will create 4 replicate staining panels for the Cyto-Trols. The remaining wells can be used for control PBMC of your choice, if desired. Add cells to the side of each well without touching pellets and let sit for 2-3 mins, then mix up and down 5-6 times using a multichannel pipette. Avoid creating bubbles.
3.4 Add 50 $\mu$l of FACS buffer to the first set of compensation control wells (F1 through G5).
3.5 Incubate 45 minutes at room temperature with gentle shaking.
3.6 Follow these steps for all wells used, including compensation controls: Add 150 $\mu$l of FACS buffer, without pipetting up and down, and spin

at 1200 rpm, 2min, @ 4˚C, for the first wash. Aspirate, resuspend in 200 μl FACS buffer & spin 1200 rpm, 2 min, @ 4˚C, for a second wash. Repeat washing with 200 μl FACS buffer for a third and fourth wash. After the 4th wash, resuspend wells in 200 μL FACS buffer, cover with foil and keep at 4˚C until ready to run on LSR II. Run within 24 hours of staining; do not fix samples.

3.7 Create a new blank experiment using baseline CST settings. Apply previously saved Application Settings for these Lyoplates, if you had saved such. Remove all parameters except the colors listed in the table below. Create a compensation specimen with these seven colors. Create tube-specific comps for each case where there are multiple comp wells for a given color (PE-Cy7, APC-H7, and Alexa647/APC).

3.8 If you did not previously save Application Settings, run the single-color controls listed below and adjust voltages to achieve specified target channels:

| COMP CONTROL | TARGET MFI |
|---|---|
| CCR7 PE | 70,000 |
| CD4 PerCP Cy5.5 | 2,500 |
| CD45RA PE Cy7 | 70,000 |
| CD38 APC | 50,000 |
| CD8 APC-H7 | 40,000 |
| CD3 V450 | 7,500 |
| HLA DR V500 | 14,000 |

3.9 Please verify that both area and height parameters are being collected for FSC and SSC.

3.10 Calculate compensation, then record the experimental samples. Name the specimens as CytoTrol_1, CytoTrol_2, etc. Please add your institution name as a keyword to all files. If using HTS, adjust settings to: Throughput mode: standard; Sample flow rate 1.0; Sample volume (ul): 200; Mixing volume (ul): 100; Mixing speed (ul/sec): 180; Number of mixes: 3; Wash volume (ul): 400. Create a small lymphocyte gate and set stopping criteria as 100,000 lymphocytes.

**Data analysis**

3.11 Analyze data with FlowJo for Mac software, if available, creating gates for the following cell subsets:

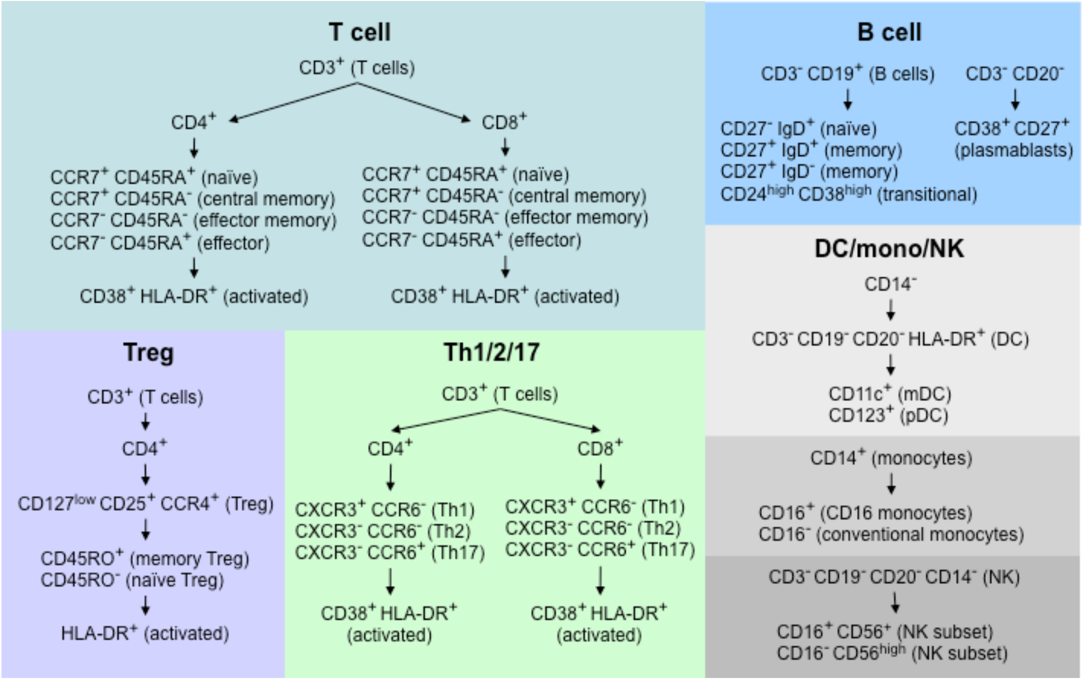


3.12 Adjust gates on a donor-specific basis, if necessary, to control for any differences in background or positive staining intensity.

3.13 Export % parent statistics for each gated population to an Excel spreadsheet. Post the spreadsheet, FlowJo workspace, and fcs files to Basecamp.

**BD Lyoplate™**

Mat No: 623882
Lot No: 35286
Exp: 2013-08-31

**Stain 80 CTT***

94 Tests
Store at 16-29°C

| | 1 | 2 | 3 | 4 | 5 | 6 | 7 | 8 | 9 | 10 | 11 | 12 | |
|---|---|---|---|---|---|---|---|---|---|---|---|---|---|
| A | CD197 PE / CD4 PerCP-Cy™5.5 / CD45RA PE-Cy™7 / CD38 APC / CD8 APC-H7 / CD3 V450 / HLA-DR V500 | | | | | | | | | | | | TCELL |
| B | CD25 PE / CD4 PerCP-Cy™5.5 / CD194 PE-Cy™7 / CD127 Alexa®Fluor647 / CD45RO APC-H7 / CD3 V450 / HLA-DR V500 | | | | | | | | | | | | TREG |
| C | CD24 PE / CD19 PerCP-Cy™5.5 / CD27 PE-Cy™7 / CD38 APC / CD20 APC-H7 / CD3 V450 / IgD V500 | | | | | | | | | | | | B CELL |
| D | CD56 PE / CD123 PerCP-Cy™5.5 / CD11c PE-Cy™7 / CD16 APC / CD3+CD19+CD20 APC-H7 / CD14 V450 / HLA-DR V500 | | | | | | | | | | | | DC/MONO/NK |
| E | CD183 PE / CD4 PerCP-Cy™5.5 / CD196 PE-Cy™7 / CD38 APC / CD8 APC-H7 / CD3 V450 / HLA-DR V500 | | | | | | | | | | | | TH1/2/17 |
| F | Neg. Beads Only | LIVE GREEN | CD197 PE | CD4 PerCP-Cy™5.5 | CD45RA PE-Cy™7 | CD194 PE-Cy™7 | CD27 PE-Cy™7 | CD11c PE-Cy™7 | CD196 PE-Cy™7 | CD38 APC | CD127 Alexa 647 | CD8 APC-H7 | COMP BEADS |
| G | CD45RO APC-H7 | CD20 APC H7 | CD3+ 19+20 APC-H7 | CD3 V450 | HLA-DR V500 | | | Neg. Beads Only | LIVE GREEN | CD197 PE | CD4 PerCP-Cy™5.5 | CD45RA PE-Cy™7 | EMPTY WELLS |
| H | CD194 PE-Cy™7 | CD27 PE-Cy™7 | CD11c PE-Cy™7 | CD196 PE-Cy™7 | CD38 APC | CD127 Alexa 647 | CD8 APC-H7 | CD45RO APC-H7 | CD20 APC H7 | CD3+ 19+20 APC-H7 | CD3 V450 | HLA-DR V500 | |

| Cocktail: | T CELL | TREG | B CELL | DC/MONO/NK | TH1/2/17 |
|---|---|---|---|---|---|
| | | | | | |
| FITC | open | open | open | open | open |
| PE | **CCR7**<br>BD 560765<br>(150503) | **CD25**<br>BD 341009<br>(2A3) | **CD24**<br>BD 555428<br>(ML5) | **CD56**<br>BD 555516<br>(B159) | **CXCR3**<br>BD 557185<br>(1C6/CXCR3) |
| PERCP-CY5.5 | **CD4**<br>BD 341654<br>(SK3) | **CD4**<br>BD 341654<br>(SK3) | **CD19**<br>BD 340951<br>(SJ25C1) | **CD123**<br>BD 558714<br>(7G3) | **CD4**<br>BD 341654<br>(SK3) |
| PE-CY7 | **CD45RA**<br>BD 337167<br>(L48) | ***CCR4****<br>*BD 557864*<br>*(1G1)* | **CD27**<br>BD 560609<br>(M-T271) | **CD11c**<br>BD 561356<br>(B-LY6) | **CCR6**<br>BD 560620<br>(11A9) |
| APC | **CD38**<br>(HIT2) | **CD127**<br>BD 560905<br>(HIL-7R-M21) | **CD38**<br>(HIT2) | **CD16**<br>BD 561304<br>(B73.1) | **CD38**<br>(HIT2) |
| APC-H7 | **CD8**<br>BD 560179<br>(SK1) | **CD45RO**<br>BD 561137<br>(UCHL1) | **CD20**<br>BD 560734<br>(2H7) | **CD3+CD19+CD20**<br>BD 560176 (SK7)<br>BD 643078 (SJ25C1)<br>BD 560734 (2H7) | **CD8**<br>BD 560179<br>(SK1) |
| V450 | **CD3**<br>BD 560365<br>(UCHT1) | **CD3**<br>BD 560365<br>(UCHT1) | **CD3**<br>BD 560365<br>(UCHT1) | **CD14**<br>BD 560349<br>(MPHIP9) | **CD3**<br>BD 560365<br>(UCHT1) |
| V500 | **HLA-DR**<br>BD 561224<br>(G46-6) | **HLA-DR**<br>BD 561224<br>(G46-6) | **IgD**<br>BD 561490<br>(IA6-2) | **HLA-DR**<br>BD 561224<br>(G46-6) | **HLA-DR**<br>BD 561224<br>(G46-6) |
